# The effects of hesperidin supplementation on insulin resistance and sensitivity in adults: a systematic review and meta-analysis of randomized controlled trials

**DOI:** 10.3389/fnut.2025.1724786

**Published:** 2026-01-09

**Authors:** Wen Li, Yuyin Liu, Kun Zhu, Lijiao Wu, Meixi Liu, Qiu Chen

**Affiliations:** 1 Department of Endocrinology, Hospital of Chengdu University of Traditional Chinese Medicine, Chengdu, China; 2 Department of Otorhinolaryngology, Hospital of Chengdu University of Traditional Chinese Medicine, Chengdu, China; 3 Department of Urology, Hospital of Chengdu University of Traditional Chinese Medicine, Chengdu, China; 4 Department of Endocrinology, Deyang Hospital Affiliated Hospital of Chengdu University of Traditional Chinese Medicine, Chengdu, China

**Keywords:** hesperidin, insulin resistance, insulin sensitivity, meta-analysis, randomized controlled trial

## Abstract

This systematic review and meta-analysis of randomized controlled trials (RCTs) aimed to evaluate the effects of hesperidin supplementation on insulin resistance and sensitivity in adults. Two authors independently searched electronic databases, including PubMed, Scopus, Embase, Web of Science, and the Cochrane Library, from their inception to July 2025 for relevant RCTs. The quality of the included studies was assessed using the Cochrane Collaboration’s tool for assessing the risk of bias. Heterogeneity among the included studies was evaluated using Cochrane’s Q test and the I^2^ statistic. A meta-analysis of eligible studies was conducted using either fixed- or random-effects models, with the weighted mean difference (WMD) serving as the overall effect size. Pre-specified subgroup and sensitivity analyses were performed to explore potential sources of heterogeneity. This meta-analysis included 16 studies, comprising 845 participants and assessing five outcome measures. We found that hesperidin supplementation significantly reduced the homeostatic model assessment of insulin resistance (HOMA-IR) (WMD: −0.43, 95%CI: −0.82, −0.03; *p* = 0.034) and increased the quantitative insulin sensitivity check index (WMD: 0.05, 95%CI: 0.01, 0.08; *p* = 0.005). However, no significant effects were observed on insulin, fasting blood glucose, or glycated hemoglobin A1c. Subgroup analyses indicated that supplementation with high-dose, long-duration purified hesperidin, particularly in individuals with metabolic disorders and obesity, had a positive effect on insulin levels. However, subsequent trial sequential analysis revealed that hesperidin confers no statistically significant improvement in HOMA-IR. In conclusion, the current body of evidence does not support hesperidin as an effective intervention for ameliorating insulin resistance or enhancing glycemic control. Future investigations are warranted to explore its potential efficacy in specific populations or subgroups.

**Systematic review registration:**

PROSPERO, identifier (CRD420251102342).

## 1 Introduction

Diabetes mellitus (DM) is a chronic endocrine disorder characterized by hyperglycemia, resulting from either insulin deficiency or insulin resistance. Globally, approximately 463 million people are affected by DM, which ranks as the ninth leading cause of death. Projections indicate that the total number of individuals with DM will reach 700 million by 2045 (1). Concurrently, the prevalence of obesity, metabolic syndrome (MetS), non-alcoholic fatty liver disease (NAFLD), and atherosclerotic cardiovascular disease is steadily increasing. This sharp rise in the prevalence of metabolic diseases is considered one of the most critical public health challenges worldwide (2). Insulin resistance (IR) is recognized as a core driver of these chronic metabolic-related conditions (3).

Insulin resistance is a prevalent clinical condition, typically defined as a pathological state in which the biological response to normal insulin concentrations is subnormal (4). It is characterized by a diminished ability of insulin to stimulate the uptake and utilization of glucose by peripheral target tissues, creating a vicious cycle. Data from the National Health and Nutrition Examination Survey suggest that approximately 40% of adults aged 18–44 exhibit IR (5), and it is present in over 90% of patients with Type 2 Diabetes Mellitus (T2DM) (6). In addition to pharmacological treatments and lifestyle modifications, such as diet and weight loss, nutritional interventions can help ameliorate metabolic disturbances. Natural compounds, valued for their efficacy and minimal side effects, represent a potential strategy for managing metabolic diseases (7).

Polyphenols, a class of bioactive compounds naturally occurring in plant-based foods, exhibit anti-inflammatory, antioxidant, and various other biological activities (8), contributing to the prevention of metabolic disorders and chronic diseases (9). Flavonoids are a major class of polyphenols and have garnered considerable attention due to their abundance in plant-based diets. Research indicates that long-term intake of flavonoid-rich foods is beneficial to human health, promotes longevity, and reduces the incidence of metabolic-related diseases (10). They are primarily categorized into six subclasses: flavonols, flavan-3-ols, flavones, flavanones, anthocyanins, and isoflavones. Hesperidin is a prominent flavanone found in citrus fruits, particularly in oranges and their juice (11). Owing to its non-cumulative nature, its consumption as a supplement or nutraceutical is considered beneficial and safe (12). Both *in vivo* and *in vitro* studies have demonstrated that hesperidin supplementation exhibits hypoglycemic effects and ameliorates parameters of insulin resistance. Given its anti-inflammatory and antioxidant properties, as well as its capacity for scavenging oxygen free radicals, increasing nitric oxide synthesis, and regulating apoptosis (13), hesperidin exerts positive effects on various conditions, including IR (14), T2DM (15), NAFLD (16), MetS (17), and cardiovascular diseases (18).

Although the mechanisms by which hesperidin improves insulin resistance have been established (19–21) and several clinical trials have assessed the impact of its supplementation on insulin resistance and glycemic-related indices, its efficacy varies across different metabolic diseases. A 2019 review by Shams-Rad et al. (22), which included six studies, found no significant effect of hesperidin on glycemic control. Subgroup analyses based on study design, health status, intervention duration, and dosage also failed to reveal any improvement in fasting blood glucose (FBG), homeostatic model assessment of insulin resistance (HOMA-IR), or insulin (INS). In the same year, Pla-Paga et al. (23) conducted a systematic review to assess the effect of hesperidin on cardiovascular risk biomarkers, including both animal and human clinical trials. They found that hesperidin supplementation reduced FBG in animal models, but no definitive conclusions were observed in clinical trials. Subsequently, several other meta-analyses have evaluated the effects of hesperidin or orange juice on cardiovascular risk factors; however, these studies were limited to either purified hesperidin or only orange juice, and their conclusions were inconsistent (24–26). Given the continued accumulation of recent randomized controlled trials (RCTs) investigating hesperidin, the differential effects across various formulations warrant clarification. Therefore, this study presents an updated systematic review and meta-analysis aimed at comprehensively evaluating the effects of hesperidin on insulin resistance and sensitivity in adults. Through subgroup analyses, we seek to identify the appropriate populations for different formulations, thereby providing more precise guidance for clinical practice and application.

## 2 Methods

The design, conduct, and reporting of this systematic review and meta-analysis adhered to the recommendations of the Preferred Reporting Items for Systematic Reviews and Meta-Analyses (PRISMA) guidelines (27) and the AMSTAR-2 quality assessment tool (28) to ensure methodological rigor and reporting completeness. The study protocol was prospectively registered with the International Prospective Register of Systematic Reviews (PROSPERO) (Registration Number: CRD420251102342).

### 2.1 Search strategy

A systematic literature search was conducted across the following electronic databases: PubMed, Scopus, Embase, Web of Science, and Cochrane Library. The search encompassed records from database inception through July 2025, with no restrictions imposed on language or publication date. We used a combination of medical subject headings and non- medical subject headings terms. The detailed search terms for all databases are provided in Supplementary Table S1. Additionally, reference lists of retrieved articles, systematic reviews, and meta-analyses were manually screened to identify other potentially eligible trials.

### 2.2 Inclusion criteria

Studies were included based on the following criteria: (1) Participants: Male or female adults (≥18 years), with no restrictions on their health status. (2) Intervention: The experimental group received hesperidin or a complex with a high hesperidin content as the intervention, with no restrictions on type or duration. (3) Comparison: The control group received a placebo or a control intervention. (4) Outcomes: Primary outcomes were HOMA-IR and quantitative insulin sensitivity check index (QUICKI). Secondary outcomes included INS, FBG, and glycated hemoglobin A1c (HbA1c). Studies reporting at least one of these outcomes were included. (5) Study design: RCTs with either a parallel or crossover design. Table 1 outlines the Population, Intervention, Comparison, Outcome, and Study design criteria for this systematic review.

**Table 1:** The population, intervention, comparison, outcome, and study type criteria.

Criteria	Description
Population	Adults age ≥ 18 years, with no restrictions on their health conditions
Intervention	hesperidin or complex formulations with a high hesperidin content (no restrictions on type or intervention duration)
Comparison	placebo or control intervention
Outcome	Outcomes of interest regarding at least one of the following variables: homeostatic model assessment of insulin resistance, quantitative insulin sensitivity check index, insulin, fasting blood glucose, glycated hemoglobin A1c
Study types	Randomized controlled clinical trials (cross-over or parallel)

### 2.3 Exclusion criteria

Studies were excluded if they met any of the following criteria: (1) evaluated the acute effects of hesperidin; (2) did not clearly report the dosage of hesperidin; (3) did not report outcomes of interest or provided incomplete information; (4) were non-randomized studies (e.g., observational studies such as cohort or case–control), animal experiments, *in vitro* studies, reviews, case reports, lectures, or conference papers; (5) RCTs lacking a placebo or control group.

### 2.4 Data extraction

Two authors (W. L. and Y. Y. L.) independently extracted data using a predefined, standardized form. The following information was extracted from each study: the first author’s name, year of publication, country of origin, study design, participant characteristics (age, sex, number of participants per group, health status), intervention characteristics (including dosage, type, and duration), outcomes, and adverse events. The mean and standard deviation (SD) of outcome measures at baseline, post-intervention, and the change from baseline were recorded. Data reported as mean ± standard error of the mean (SEM) were converted to mean ± SD using the formula SEM = SD/ $\sqrt{N}$ . For studies that reported the median and the corresponding first and third quartiles, we estimated the sample mean and SD using the formula proposed by Wan et al. (29). If the SD of the mean difference was not provided in the publication, it was calculated using the following formula: $\text{SDchange}=\sqrt{{\left(\text{SDbaseline}\right)}^{2}+{\left(\text{SDfinal}\right)}^{2}-(2\times R\times \text{SDbaseline}\times \text{SDfinal})}$ (30). For crossover trials, paired analysis data of differences were preferentially extracted. If a study included multiple intervention periods, data from the longest period were extracted. If a study included multiple intervention groups, data from the group most directly relevant to hesperidin intervention alone were extracted. Cross-checking was performed following data extraction, and any discrepancies were resolved through discussion with a third reviewer (QC). Prior to calculating the effect size, serum glucose values were converted to mg/dL, serum insulin to μU/mL, and hesperidin dosage to mg/d.

### 2.5 Quality assessment

The Cochrane Risk of Bias 2 (RoB 2) tool was used to assess the risk of bias in the included studies. The assessment covered five domains: the randomization process, deviations from intended interventions, missing outcome data, measurement of the outcome, and selection of the reported result. Ultimately, an overall risk of bias was determined for each study. For each RCT, two reviewers (W. L. and Y. Y. L.) independently evaluated each domain, assigning a risk level of “low risk,” “some concerns,” or “high risk.” The results were cross-checked, and any disagreements were resolved by consulting a third reviewer (Q. C.).

### 2.6 Certainty assessment

The overall quality of evidence for included studies was evaluated and summarized using the Grading of Recommendations, Assessment, Development and Evaluation (GRADE) system (31). The quality of evidence was categorized into four levels: very low, low, moderate, and high.

### 2.7 Data integration and statistical analysis

We used either fixed- or random-effects models to estimate the overall effect size, presenting the weighted mean difference (WMD) and the 95% confidence interval (CI) in a forest plot. Heterogeneity among the included studies was assessed using Cochrane’s Q test and the I-square (I^2^) statistic; significant heterogeneity was considered present if *I*^2^ > 50% or *p* < 0.1 (32). To explore sources of heterogeneity, we conducted subgroup analyses to evaluate whether the results differed by the following factors: study type, hesperidin dosage, hesperidin type, intervention duration, participants’ health status, baseline body mass index (BMI), lifestyle modification, and the study’s risk-of-bias quality. A leave-one-out sensitivity analysis was performed to assess the stability of the results. To specifically investigate the influence of lifestyle interventions and hesperidin formulation types on the primary findings, subgroup-specific sensitivity analyses were additionally conducted. We visually inspected funnel plots and used Begg’s rank correlation test and Egger’s linear regression test to evaluate potential publication bias (33). If publication bias was detected, the trim-and-fill method was used to estimate the potential impact of unpublished studies (34). We applied fractional polynomial modeling to further investigate potential non-linear dose–response relationships between hesperidin supplementation (mg/d) and treatment duration (weeks) and the relevant outcomes.

To evaluate the reliability of the current meta-analysis conclusions and control for the risk of random error, trial sequential analysis (TSA) was performed for the primary outcome (HOMA-IR). The type I error rate (α) was set at 5% (two-sided), with a statistical power (1 − β) of 80%. The required information size (RIS) was calculated based on the observed mean difference in the control group using variance estimation methods. If the cumulative Z-curve crossed the conventional significance boundary (*p* = 0.05) but failed to cross the TSA monitoring boundary or reach the RIS, this was interpreted as insufficient evidence, suggesting a potentially false-positive conclusion. Conversely, if the Z-curve crossed the TSA boundary and reached the RIS, this indicated that the existing evidence was sufficient and the conclusion robust. All statistical analyses were performed using STATA 15.0, TSA software (version 0.9.5.10 Beta), and Anaconda. Statistical significance was defined as *p* < 0.05.

## 3 Results

### 3.1 Study selection

**Figure 1:**
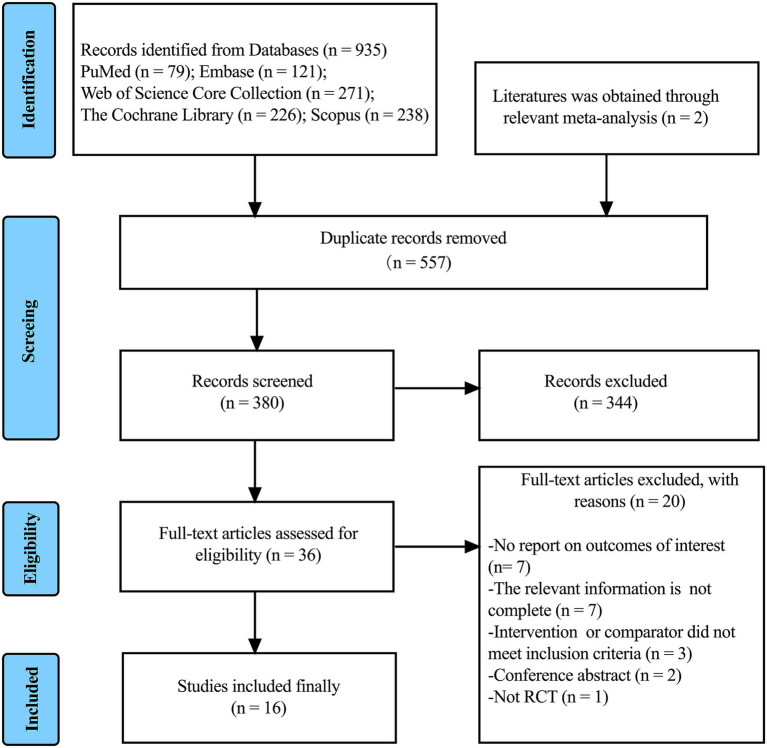
Flowchart of the literature screening process.

A total of 935 articles were retrieved from the five databases, and two additional articles were identified through manual searches of other relevant literature. After removing 557 duplicate records and excluding 344 clearly irrelevant studies based on title and abstract screening, 36 articles were assessed for full-text eligibility. Following this screening, 16 studies were ultimately included in the meta-analysis (17, 35–49). Twenty studies were excluded for reasons detailed in Supplementary Table S2. A detailed flowchart of the selection process is presented in Figure 1.

### 3.2 Study characteristics

This analysis included 16 RCTs, encompassing a total of 845 participants. The studies were published between 2010 and 2025 and were conducted in countries including the United Kingdom, France, Italy, the Netherlands, Iran, Brazil, Egypt, and Spain. Overall, there were 12 parallel and 4 crossover studies. All crossover studies reported washout periods ranging from 3 days to 7 weeks, with three studies confirming the absence of carryover effects through statistical testing. The participants included healthy but overweight individuals, as well as patients with obesity, T2DM, MetS, NAFLD, and individuals with depressive symptoms following Coronary Artery Bypass Graft surgery. The number of participants ranged from 18 to 100. The duration of the interventions varied from 3 to 12 weeks, and the daily dosage of hesperidin supplementation ranged from 61.7 mg to 1,000 mg. Ten studies used purified hesperidin capsules as the intervention, while six studies used a hesperidin complex (including orange juice or citrus flavonoid supplements). Seven studies incorporated concurrent lifestyle modifications (dietary and exercise interventions). Dietary interventions included very-low-calorie diets, balanced diets, and Mediterranean diets, whereas exercise protocols consistently prescribed 150 min per week of moderate-intensity physical activity. Only one study (38) reported an adverse event: one patient developed a skin rash, which resolved after discontinuing the supplement. Regarding funding sources, 13 studies received external financial support from government agencies, universities, industry sponsors (including citrus-related companies), and non-profit organizations. Four industry-funded studies explicitly stated that the sponsors had no involvement in critical aspects of the research, including data collection, analysis, interpretation, or publication decisions. Fifteen studies declared no conflicts of interest, while one study disclosed author affiliations with external companies. Overall, the included studies demonstrated adequate transparency in disclosing funding sources and potential conflicts of interest. The detailed characteristics of the included studies are presented in Table 2.

**Table 2:** Basic information of the literature studies.

Study	Country	Design	Number, sex (M/F)	Mean age (year)	Mean BMI (kg/m^2^)	Duration (week)	Intervention	Control	Variables presented	Notes about participants	Funding information and conflict of interest
Morand et al. (35)	France	Crossover	24 (M)	56	27.4	4 + 3 wk. washout period	500 mL/d control drink plus 292 mg/d hesperidin	500 mL/d control drink plus placebo (starch)	INS, FBG	Healthy overweight men	Funding: Florida Department of Citrus Role: Funder declared no role in design, analysis, or publication COI: No competing interests
Rizza et al. (17)	Italy	Crossover	24 (15/9)	52	34.7	3 + day washout period	500 mg/d hesperidin capsule	Placebo capsule (cellulose)	QUICKI, INS, FBG, HbA1c	Patients with MetS	Funding: NIH Intramural Program; University of Tor Vergata grant; Blue California material gift* COI: No competing interests
Simpson et al. (36)	UK	Parallel	Int: 18 (M) Con: 18 (M)	Int: 48.3 Con: 48.9	Int: 29.9 Con: 29.3	12	250 mL/d OJ (hesperidin: 135.4 mg/d)	250 mL/d control drink (hesperidin: 0)	HOMA-IR	Healthy overweight men	Funding: Florida Department of Citrus (provided funding and orange juice) Role: Approved study design but had no involvement in data collection, analysis, interpretation, or publication decisions COI: IAM is on Scientific Advisory Boards for Nestlé, Ikea and Mars Inc.
Sweidan (37)	UK	Crossover	23 (19/4)	37	30	4 + 4 wk. washout period	Citrus flavonoid supplement (hesperidin: 140 mg/d)	Placebo	FBG	Healthy overweight individuals	Funding: Scholarship from the Ministry of Higher Education of Libya and The University of Al-Jabal Al-Gharbi* COI: No competing interests
Salden et al. (38)	Netherlands	Parallel	Int: 34 (17/17) Con: 34 (12/22)	Int: 54 Con: 53	Int: 28.2 Con: 29.7	6	450 mg/d hesperidin 2S capsule	Placebo capsule (cellulose)	QUICKI, INS, FBG	Healthy overweight individuals	Funding: BioActor BV Role: The funder had no involvement in any aspect of the research or publication COI: No competing interests
Homayouni et al. (39)	Iran	Parallel	Int: 31 (14/17) Con: 29 (14/15)	Int: 51.26 Con: 54.41	Int: 27.97 Con: 27.49	6	500 mg/d hesperidin capsule	Placebo capsule (starch)	HOMA-IR, FBG	Patients with T2DM	Funding: Nutrition and Metabolic Disease Research Center, Ahvaz Jundishapur University of Medical Sciences (NRC-9411)* COI: No competing interests
Ribeiro et al. (40)	Brazil	Parallel	Int: 39 Con: 39	Int: 37 Con: 35	Int: 33 Con: 34	12	500 ml/d of OJ (hesperidin: 162 mg/d) with a reduced-calorie diet	A reduced-calorie diet	HOMA-IR, INS, FBG	Patients with obesity	Funding: PADC/FCFAr-UNESP; Citrosuco S. A; CAPES scholarship* COI: No competing interests
Cheraghpour et al. (41)	Iran	Parallel	Int: 25 (10/13) Con: 24 (12/12)	Int: 47.32 Con: 47.29	Int: 31.7 Con: 33	12	1,000 mg/d hesperidin capsule plus lifestyle modification program	Placebo capsule (starch) plus lifestyle modification program	HOMA-IR, INS, FBG	Patients with NAFLD	Funding: NR COI: No competing interests
Ponce et al. (42)	Brazil	Parallel	Int: 36 (12/14) Con: 36 (11/25)	Int: 49 Con: 46	Int: 34 Con: 35.1	12	500 ml/d OJ (hesperidin: 121.6 mg/d) plus balanced diet	balanced diet	HOMA-IR, INS, FBG	Patients with MetS	Funding: CitrusBR CAPES, Citrosuco S. A. Role: The sponsors had no role in the study’s design, execution, interpretation, or writing COI: No competing interests
Yari et al. (43)	Iran	Parallel	Int: 25 (12/13) Con: 24 (13/11)	Int: 45.05 Con: 45.33	Int: 29.63 Con: 32.93	12	1,000 mg/d hesperidin capsule plus lifestyle modification program	Placebo capsule (starch) plus lifestyle modification program	HOMA-IR, QUICKI, INS, FBG	Patients with MetS	Funding: Shahid Beheshti University of Medical Sciences, Tehran, Iran (Grant No. 1397/61461)* COI: No competing interests
Yari et al. (44)	Iran	Parallel	Int: 22 (11/11) Con: 21 (10/11)	Int: 45.82 Con: 46.11	Int: 31.07 Con: 33.06	12	1,000 mg/d hesperidin capsule plus lifestyle modification program	lifestyle modification program	HOMA-IR, QUICKI, INS, FBG	Patients with NAFLD	Funding: NR COI: No competing interests
Yari et al. (45)	Iran	Parallel	Int: 22 (12/10) Con: 22 (11/11)	Int: 45.82 Con: 46.41	Int: 29.97 Con: 33.09	12	1,000 mg/d hesperidin capsule plus lifestyle modification program	lifestyle modification program	HOMA-IR, QUICKI, INS, FBG	Patients with MetS	Funding: Shahid Beheshti University of Medical Sciences, Tehran, Iran (Grant No. 1397/61463)* COI: No competing interests
Booyani et al. (46)	Iran	Parallel	Int: 36 (25/11) Con: 32 (25/7)	Int: 59.75 Con: 58.56	Int: 27.25 Con: 25.90	12	200 mg/d hesperidin capsule	Placebo capsule (starch)	FBG	Patients with depression after CABG	Funding: Iran University of Medical Sciences, (Grant/Award Number: 95-04-27-29848)* COI: No competing interests
Osama et al. (47)	Egypt	Parallel	Int: 32 (15/17) Con: 33 (16/17)	Int: 50.6 Con: 51.8	Int: 27.53 Con: 28.31	12	1,000 mg/d hesperidin capsule plus antidiabetic drug	Antidiabetic drug	FBG	Patients with T2DM	Funding: None COI: No competing interests
Navajas-Porras et al. (48)	Spain	Parallel	Int: 22 (11/11) Con: 20(7/13)	Int: 49.8 Con: 48.6	Int: 40.3 Con: 38.2	6	200 mL/d OJ (hesperidin: 143.8 mg/d) with a reduced-calorie diet	200 ml/d placebo Juice (hesperidin: 61.7 mg/d) with a reduced-calorie diet	HOMA-IR, INS, FBG, HbA1c	Patients with obesity	Funding: Carlos III Health Institute (ISCIII) (PI24/01010); European Regional Development Fund (PI21/001160); Generalitat Valenciana Ministry of Education (CIPROM/2022/32)* COI: No competing interests
Rangel-Huerta et al. (49)	Spain	Crossover	100	NR	Int: 33.2 Con: 33.1	12 + 7 wk. washout period	500 ml/d high-polyphenol OJ (hesperidin: 582.5 mg/d)	500 ml/d regular-polyphenol OJ (hesperidin: 237.5 mg/d)	HOMA-IR, INS, FBG	Patients with obesity/ overweight	Funding: University of Granada and Coca Cola Europe (Contract 3,345)* COI: No competing interests

M, male; F, female; BMI, body mass index; Int, intervention; Con, control; d, day; wk, week; OJ, orange juice; HOMA-IR, homeostatic model assessment of insulin resistance; QUICKI, quantitative insulin sensitivity check index; INS, insulin; FBG, fasting blood glucose; HbA1c, glycated hemoglobin; MetS, metabolic syndrome; T2DM, type 2 diabetes; NAFLD, nonalcoholic fatty liver disease; CABG, coronary artery bypass graft; NR, not reported; COI, conflict of interest; None, No external funding was received; *, Funder’s role not stated.

### 3.3 Quality assessment

**Figure 2:**
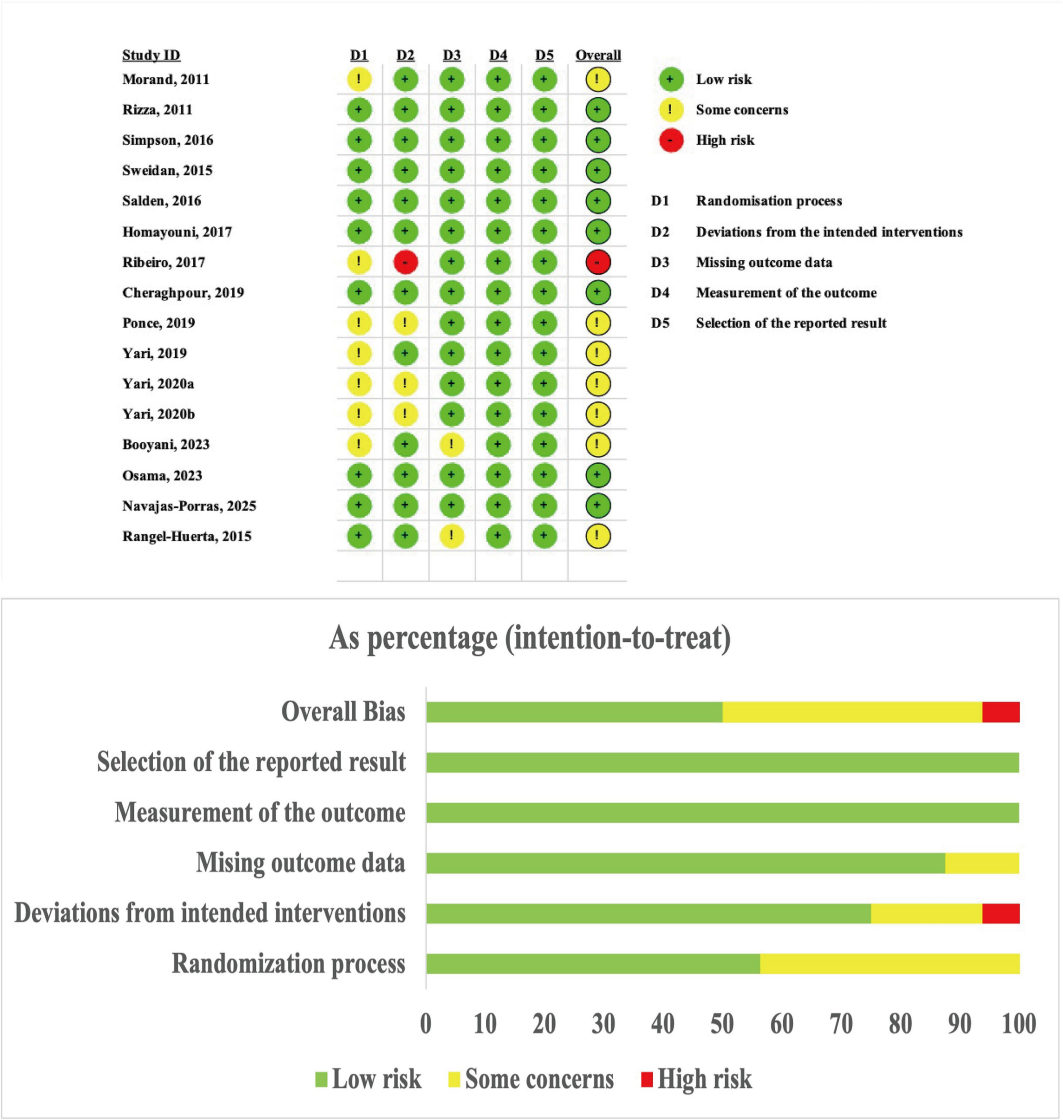
Visualization and traffic light plots of the risk of bias assessment using RoB 2.

We assessed the risk of bias in the 16 included studies using the Cochrane RoB 2 tool (50). Although 50% of the studies were rated as having a low risk of bias, 43% had some concerns, and 7% had a high risk of bias. Across all studies, the domains of “measurement of the outcome” and “selection of the reported result” were consistently rated as low risk. Seven studies did not specify their allocation concealment method, leading to an increased risk in the randomization process. Four studies were not blinded, which explains the “some concerns” rating in the “deviations from intended interventions” domain. One study had a high risk of bias due to the high recognizability of its intervention and the failure to use an intention-to-treat analysis. In two studies, high dropout rates and the lack of handling for missing data increased the risk in the “missing outcome data” domain. The RoB 2 visualization and traffic light plots for the risk of bias assessment are shown in Figure 2.

### 3.4 Results of the meta-analysis

#### 3.4.1 Effect of hesperidin supplementation on HOMA-IR and subgroup analyses

**Figure 3:**
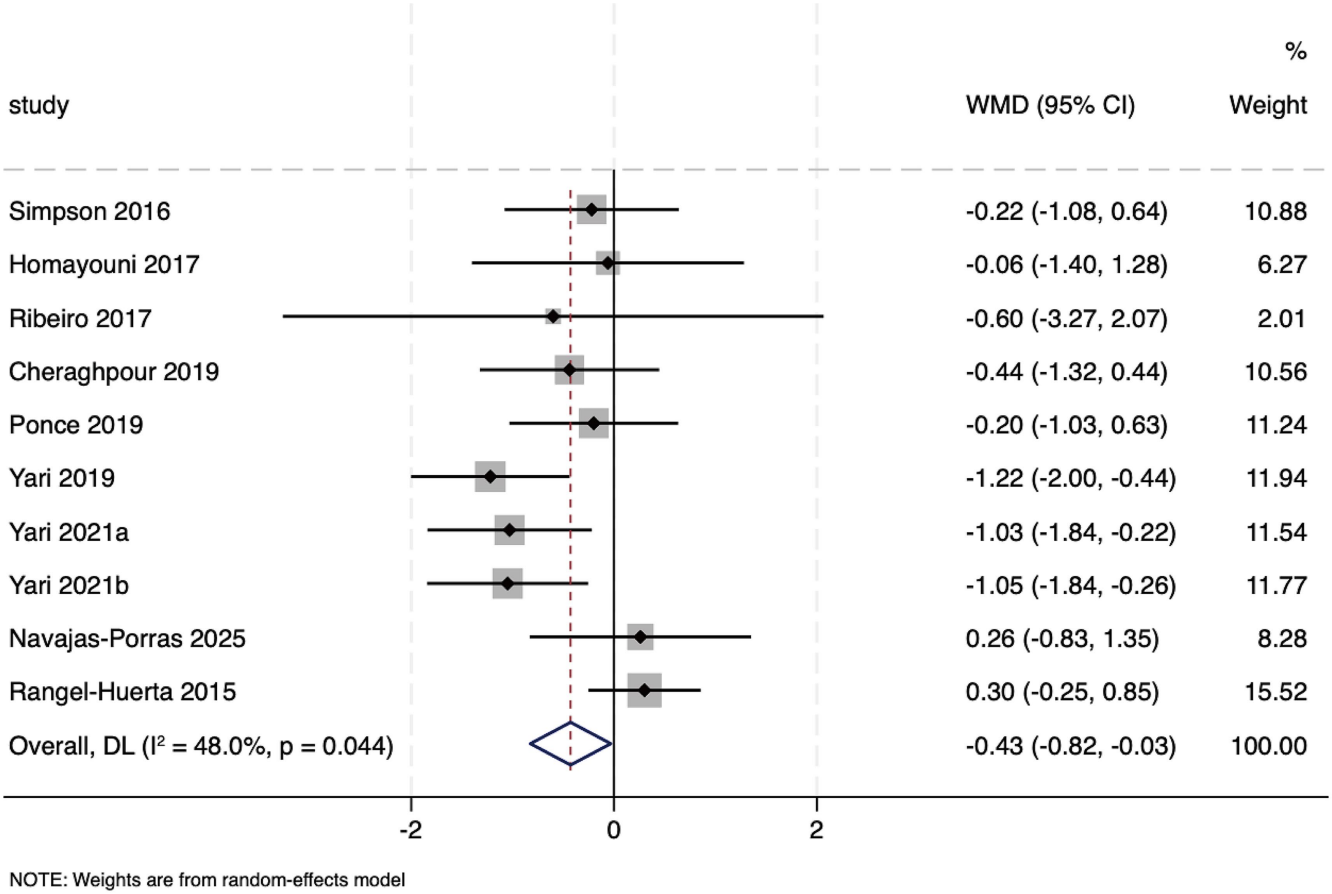
Forest plot of the HOMA-IR.

Ten studies reported the effect of hesperidin supplementation on HOMA-IR. The test for heterogeneity indicated moderate heterogeneity among these studies (*I*^2^ = 48%, *p* = 0.044 for the Q-test). Consequently, a random-effects model was used for the meta-analysis. The pooled effect size from the random-effects model revealed a significant effect of hesperidin supplementation on HOMA-IR (WMD: −0.43, 95%CI: −0.82, −0.03; *p* = 0.034) (Figure 3). Subgroup analyses showed that the HOMA-IR lowering effect was more pronounced at hesperidin dosages > 500 mg/d (WMD: −0.52, 95%CI: −0.85, −0.19; *p* = 0.002), with intervention durations > 6 weeks (WMD: −0.45, 95%CI: −0.74, −0.16; *p* = 0.002), and with the supplementation of purified hesperidin (WMD: −0.89, 95%CI: −1.28, −0.50; *p* < 0.001). Furthermore, HOMA-IR levels were significantly reduced after hesperidin supplementation in individuals with metabolic abnormalities (WMD: −0.41, 95%CI: −0.69, −0.12; *p* = 0.005), those with a baseline BMI ≥ 30 kg/m^2^ (WMD: −0.43, 95%CI: −0.72, −0.13; *p* = 0.004), populations that incorporated lifestyle modification during the intervention (WMD: −0.70, 95%CI: −1.05, −0.36; *p* < 0.001), and in studies with a parallel design (WMD: −0.61, 95%CI: −0.92, −0.30; *p* < 0.001). Notably, the reducing effect of hesperidin was more significant in studies with a higher risk of bias (WMD: −0.48, 95%CI: −0.81, −0.16; *p* = 0.003) (Supplementary Table S3). The TSA results for HOMA-IR demonstrated that the cumulative Z-curve crossed the RIS without reaching either the conventional significance boundary or the TSA monitoring boundary (Supplementary Figure S1). This indicates that even with continued sample size accrual, a statistically significant beneficial conclusion is unlikely to be attained at the currently observed effect magnitude, suggesting that the significance detected in the conventional meta-analysis may be susceptible to false-positive risk.

#### 3.4.2 Effect of hesperidin supplementation on the QUICKI and subgroup analysis

**Figure 4:**
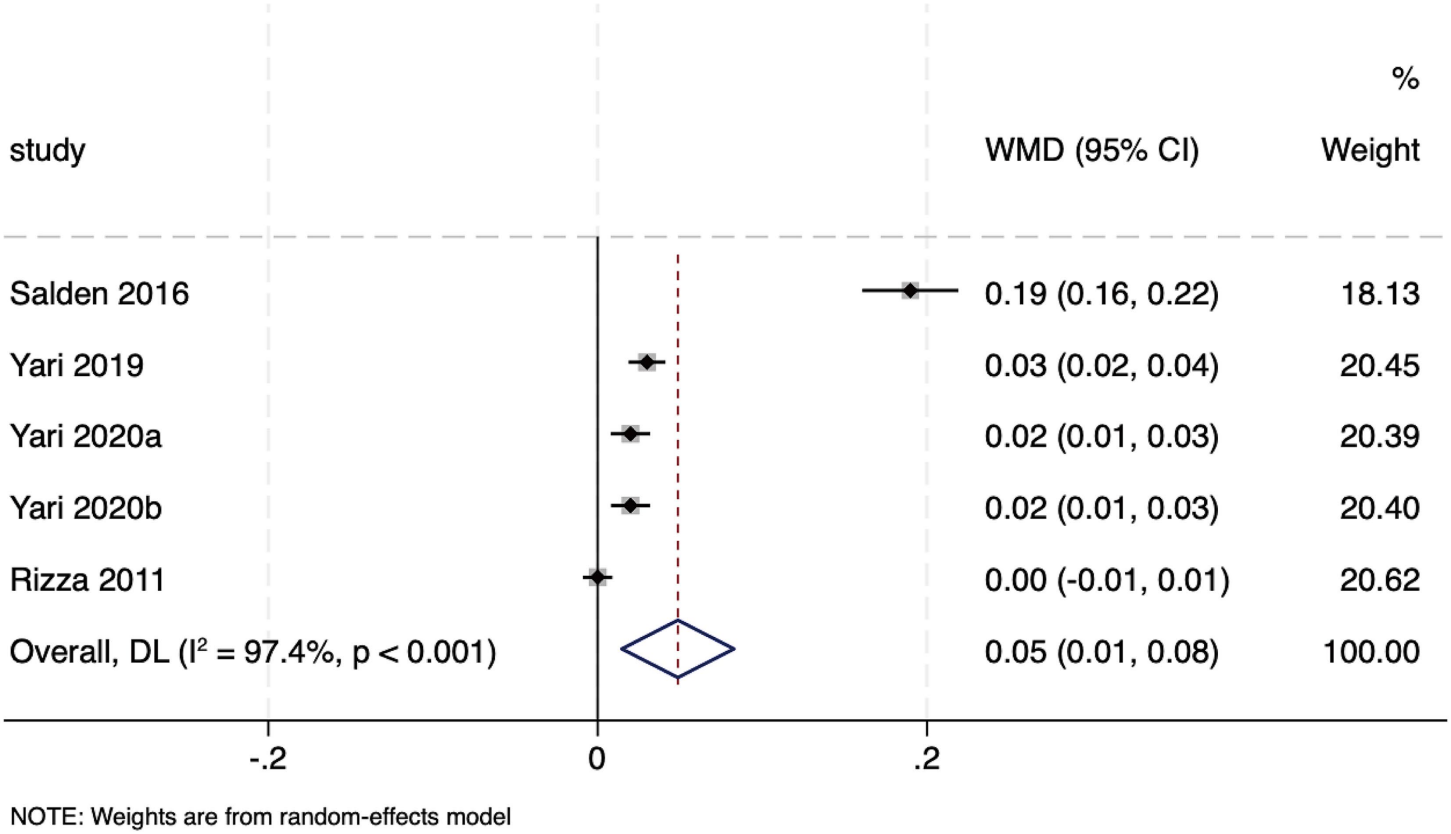
Forest plot of the QUICKI.

The pooled analysis of five trials revealed that hesperidin supplementation had a significant effect on the QUICKI (WMD: 0.05, 95%CI: 0.01, 0.08; *p* = 0.005) (Figure 4). However, substantial heterogeneity was observed among the studies (*I*^2^ = 97.4%, *p* < 0.001). Subgroup analyses were conducted to explore potential sources of this heterogeneity. The beneficial effect of hesperidin was enhanced in individuals without metabolic abnormalities and with lower BMI (BMI < 30) (WMD: 0.19, 95% CI: 0.16, 0.22; *p* < 0.001), whereas the effect size was attenuated in those with metabolic disorders and BMI ≥ 30 (WMD: 0.02, 95% CI: 0.01, 0.02; *p* < 0.001). Furthermore, the effect on QUICKI was more stable in parallel-group trials (WMD: 0.03, 95%CI: 0.03, 0.04; *p* < 0.001); the effect in crossover trials was not statistically significant, as it was based on a single study. Concurrent lifestyle modification did not significantly alter the effect size; heterogeneity was extremely high in the subgroup without lifestyle modification (*I*^2^ = 99.3%) but was absent in the group with such adjustments (*I*^2^ = 0%) (Supplementary Table S4).

#### 3.4.3 Effect of hesperidin supplementation on INS and subgroup analysis

**Figure 5:**
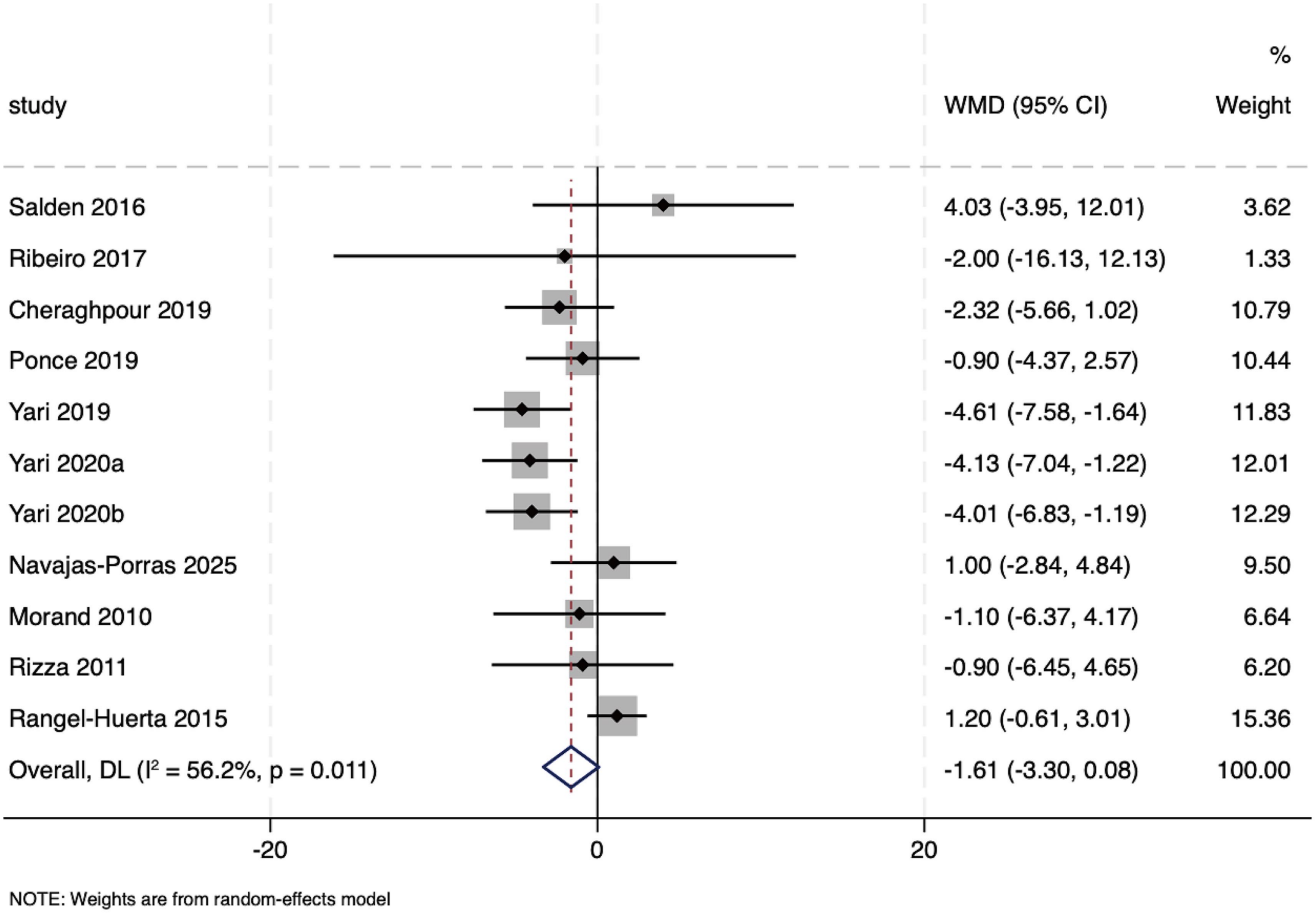
Forest plot of the INS.

Eleven studies reported the effect of hesperidin supplementation on INS. Heterogeneity testing indicated the presence of heterogeneity among these studies (*I*^2^ = 56.2%, *p* = 0.011). The pooled effect size, calculated using a random-effects model, showed that hesperidin supplementation did not have a significant effect on INS (WMD: −1.61 μU/mL, 95%CI: −3.30, 0.08; *p* = 0.062) (Figure 5). Subgroup analyses revealed that a significant reduction in INS was achieved with the use of high-concentration purified hesperidin (WMD: −3.25, 95%CI: −4.63, −1.88; *p* < 0.001), a high dosage (>500 mg/d) (WMD: −1.80, 95%CI: −2.95, −0.65; *p* = 0.002), and a longer duration of intervention (>6 weeks) (WMD: −1.71, 95%CI: −2.80, −0.62; *p* = 0.002). Other subgroup analyses found that hesperidin improved INS when combined with lifestyle modification such as diet and exercise (WMD: −2.89, 95%CI: −4.18, −1.60; *p* < 0.001) and in studies with a parallel-group design (WMD: −2.71, 95%CI: −3.98, −1.44; *p* < 0.001). This effect was also observed in patients with metabolic disorders, particularly in those with a BMI exceeding 30 (WMD: −1.49, 95%CI: −2.52, −0.46; *p* = 0.005). Moreover, similar to the findings for HOMA-IR, a significant effect was only apparent in studies with a medium to high risk of bias (WMD: −1.62, 95%CI: −2.74, −0.49; *p* = 0.005) (Supplementary Table S5).

#### 3.4.4 Effect of hesperidin supplementation on FBG and subgroup analysis

**Figure 6:**
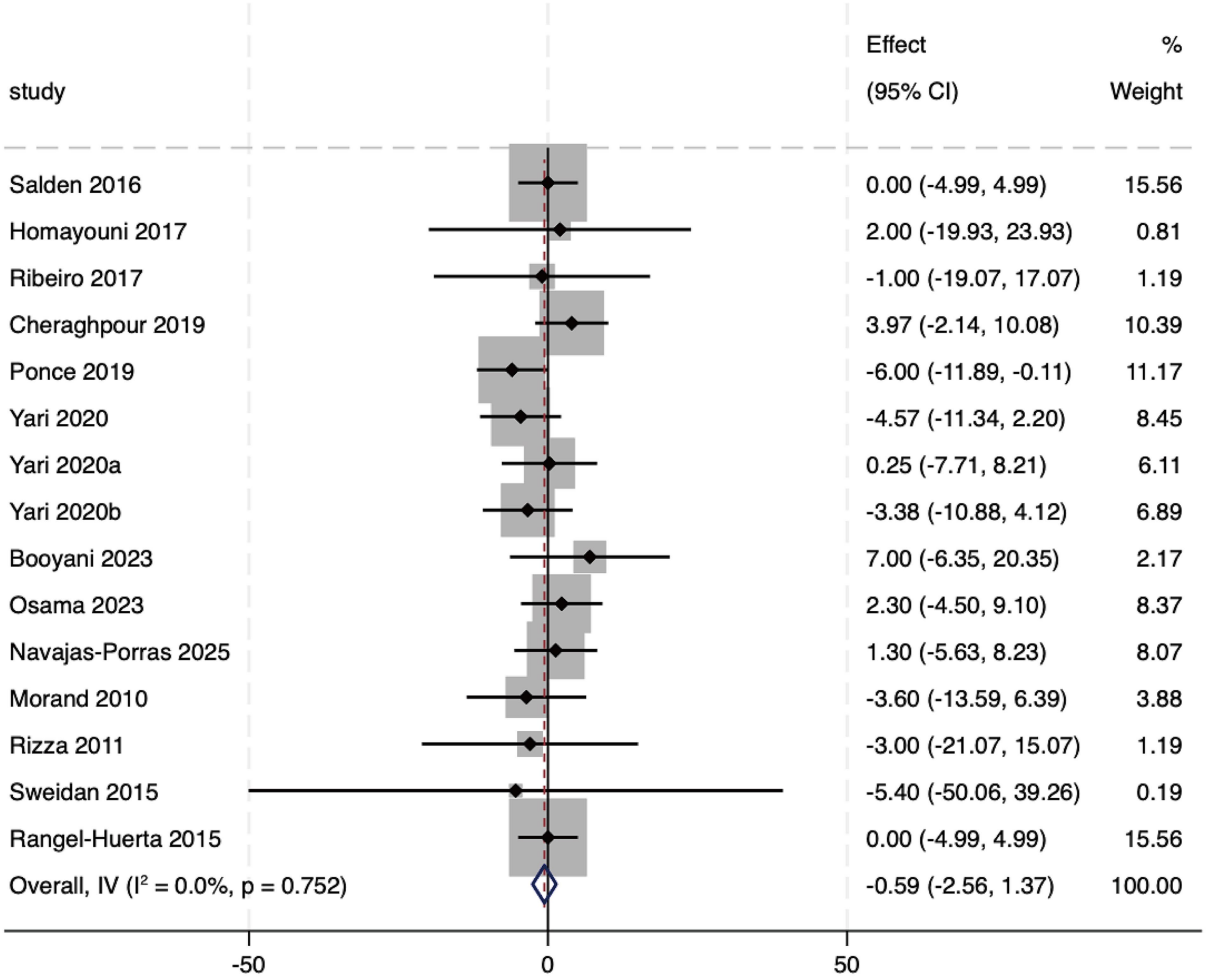
Forest plot of the FBG.

A comprehensive analysis of 15 included studies showed that, compared to the control group, hesperidin supplementation did not significantly reduce FBG levels (WMD: −0.59 mg/dL, 95%CI: −2.56, 1.37; *p* = 0.555), with no heterogeneity detected among the studies (*I*^2^ = 0%, *p* = 0.752) (Figure 6). Furthermore, subgroup analyses based on different intervention types, dosages, durations, study designs, baseline health conditions, and accompanying lifestyle modification also observed no significant differences (Supplementary Table S6). The extremely low heterogeneity suggests a high degree of consistency in these findings.

#### 3.4.5 Effect of hesperidin supplementation on HbA1c

**Figure 7:**
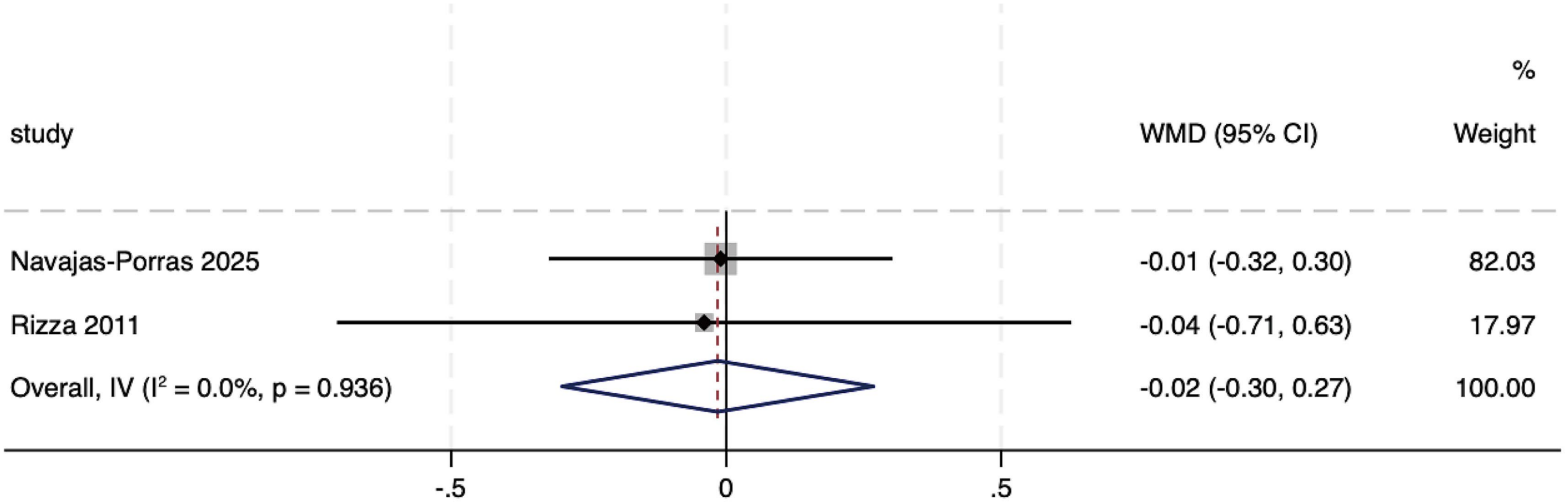
Forest plot of the HbA_1_c.

Two studies reported the effect of hesperidin supplementation on HbA1c. The heterogeneity test indicated no heterogeneity between the studies (*I*^2^ = 0%, *p* = 0.936). A fixed-effect meta-analysis showed that hesperidin supplementation did not have a significant effect on HbA1c (WMD: −0.02, 95%CI: −0.30, 0.27; *p* = 0.915) (Figure 7). Owing to the limited number of included studies, sensitivity analysis and assessment of publication bias were not performed.

### 3.5 Non-linear dose–response analysis

**Figure 8:**
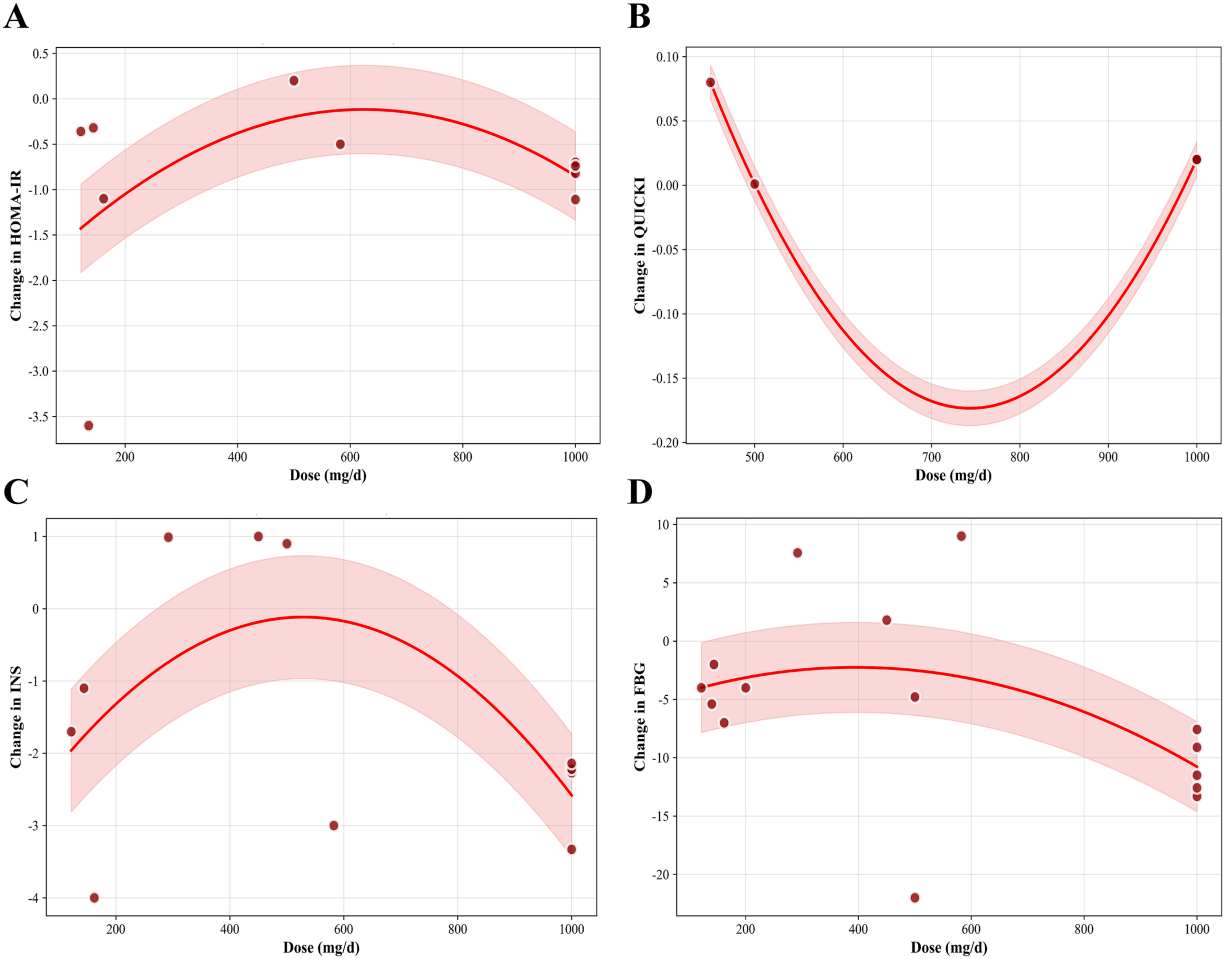
Non-linear dose–response analysis on effects of hesperidin dosage (mg/d) on **(A)** HOMA-IR, **(B)** QUICKI, **(C)** INS, **(D)** FBG.

**Figure 9:**
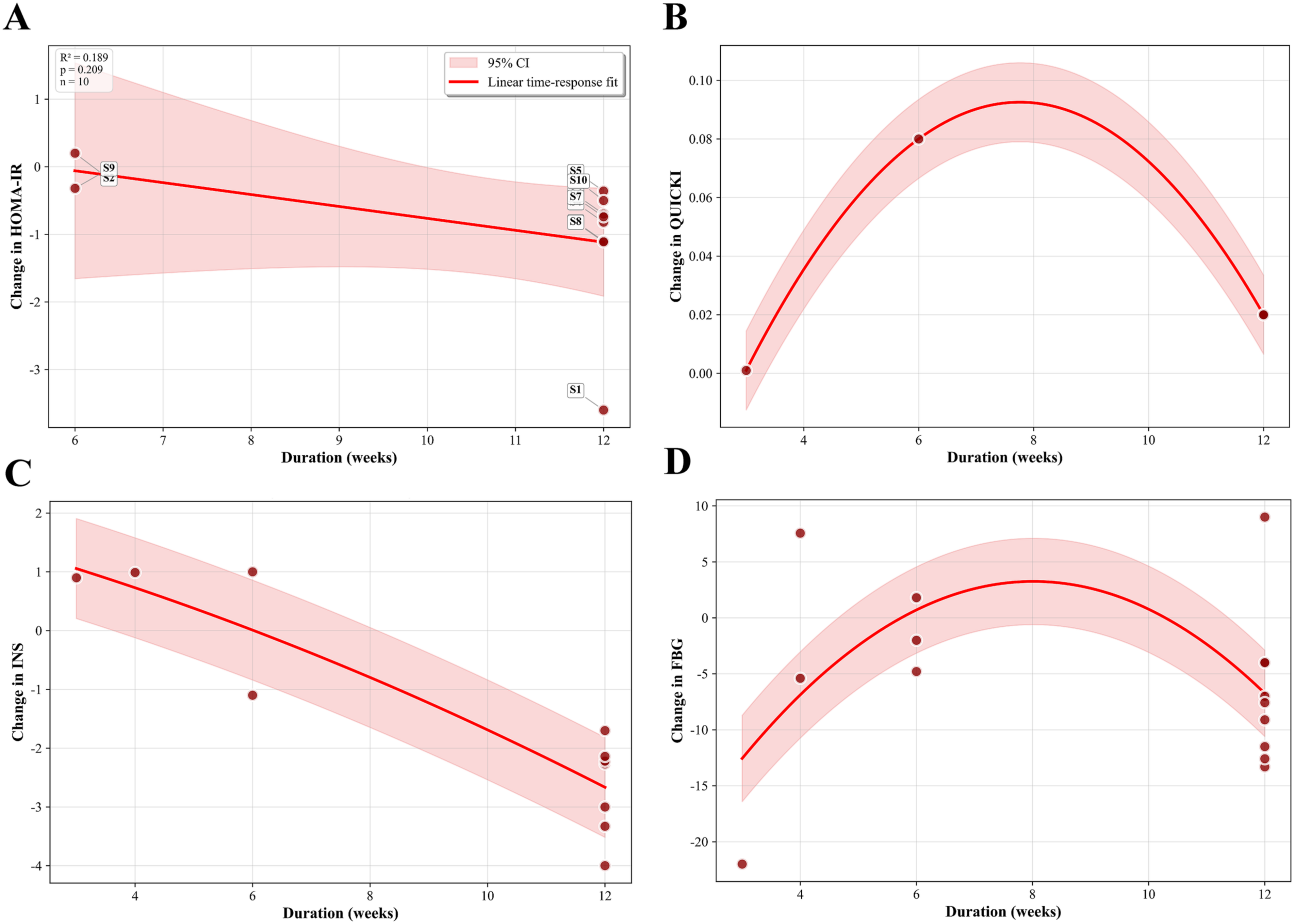
Non-linear dose–response analysis on effects of duration of the intervention (week) on **(A)** HOMA-IR, **(B)** QUICKI, **(C)** INS, **(D)** FBG.

We employed a non-linear dose–response regression model to investigate the dose–response relationship between hesperidin supplementation and indices of insulin resistance. The results revealed a non-linear relationship between hesperidin dosage (mg/d) and QUICKI (*P*_non-linearity_ < 0.001) (Figure 8B). The curve exhibited a U-shape, where the magnitude of improvement increased steadily with the dosage in the 700–1,000 mg/d range, reaching a maximum within this higher dosage range at 1,000 mg/d. Additionally, an inverted U-shaped relationship was observed between the intervention duration and QUICKI (*P*_non-linearity_ < 0.001) (Figure 9B). The effect peaked at 6 weeks and stabilized to an optimal level as the duration extended to 10–12 weeks. A non-linear relationship was also present between the intervention duration and INS (*P*_non-linearity_ = 0.001), with the most significant reduction occurring at 10–12 weeks (Figure 9C). However, we did not observe a significant non-linear effect of dosage on HOMA-IR (*P*_non-linearity_ = 0.463) (Figure 8A), INS (*P*_non-linearity_ = 0.183) (Figure 8C), or FBG (*P*_non-linearity_ = 0.219) (Figure 8D). Similarly, the duration of the intervention did not have a significant non-linear relationship with HOMA-IR (*P*_non-linearity_ = 0.209) (Figure 9A) or FBG (*P*_non-linearity_ = 0.253) (Figure 9D).

### 3.6 Sensitivity analysis

The results of the sensitivity analysis indicated that the effect sizes for the impact of hesperidin supplementation on HOMA-IR, QUICKI, and FBG were robust to a leave-one-out analysis. Furthermore, excluding the study by Ribeiro et al., which was identified as having a high risk of bias, did not alter the results. However, the analysis for INS was sensitive to the findings of Rangel-Huerta et al. (WMD: −2.37, 95%CI: −3.78, −0.95) and Salden et al. (WMD: −1.82, 95%CI: −3.52, −0.12); exclusion of either study rendered the overall pooled result statistically significant. Notably, for FBG, the removal of the study by Ponce et al. resulted in a positive WMD, a direction of effect opposite to that of the other studies. Nevertheless, this exclusion did not substantively change the statistical significance of the pooled effect size, suggesting that the results of this meta-analysis possess good robustness (Supplementary Table S7).

Further subgroup-specific sensitivity analyses indicated that lifestyle modification represents an important effect modifier. When all studies incorporating concurrent lifestyle modification were excluded, the beneficial effect of hesperidin on HOMA-IR was no longer significant (WMD: 0.12, 95% CI: −0.32, 0.56; *p* = 0.578), whereas analysis restricted to this subgroup alone yielded a significant effect (WMD: −0.68, 95% CI: −1.08, −0.28; *p* = 0.001). Similarly, intervention type significantly influenced the results; when only studies using purified hesperidin supplements were included, significant reductions in both HOMA-IR and INS were observed, whereas no significant effects were detected in the hesperidin compound subgroup (Supplementary Table S8). These findings suggest that the primary conclusion regarding hesperidin-mediated improvement in insulin resistance is substantially influenced by the inclusion of studies with lifestyle modification, and that the INS-lowering effect is predominantly attributable to purified hesperidin formulations.

### 3.7 Publication bias

**Figure 10:**
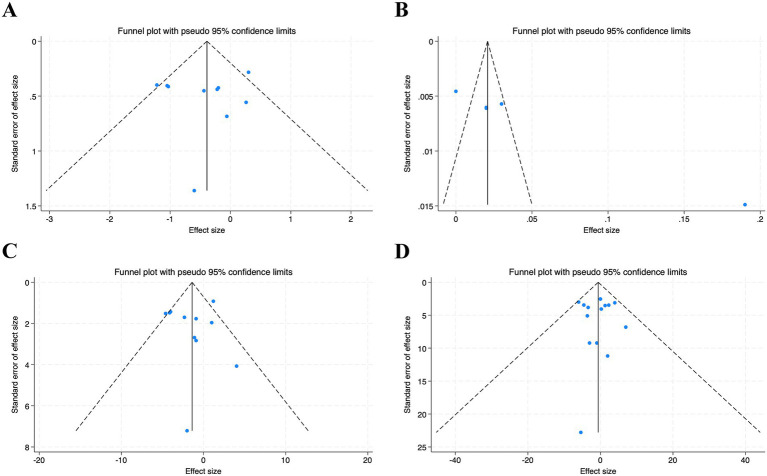
Funnel plots for the effect of hesperidin consumption on **(A)** HOMA-IR, **(B)** QUICKI, **(C)** INS, **(D)** FBG.

Visual inspection of the funnel plots revealed that they were largely symmetrical, indicating no obvious publication bias. This was supported by formal statistical testing, as neither Begg’s test nor Egger’s test showed significant publication bias for the effects of hesperidin supplementation on HOMA-IR (Begg’s *p* = 0.421; Egger’s *p* = 0.861), INS (Begg’s *p* = 0.392; Egger’s *p* = 0.629), or FBG (Begg’s *p* = 0.805; Egger’s *p* = 0.750). Although visual inspection of the funnel plot for QUICKI suggested some asymmetry and Egger’s test was significant (*p* = 0.006), Begg’s test did not indicate significant publication bias (*p* = 0.142). After applying the trim and fill method to adjust for potential publication bias, no missing studies were imputed, and the pooled effect size remained unchanged. This suggests that the results of this meta-analysis were not markedly affected by publication bias. An assessment of publication bias for HbA1c was not conducted owing to the small number of included studies. The results are detailed in Figure 10.

### 3.8 GRADE assessment

The GRADE framework was used to assess the overall quality of evidence for the five outcomes in this meta-analysis. According to the GRADE criteria, the quality of evidence was rated as moderate for FBG, low for HOMA-IR and INS, and very low for QUICKI and HbA1c. This indicates that more rigorously designed clinical trials are required in the future to corroborate our findings (Table 3).

**Table 3:** GRADE profile of effect of hesperidin supplementation on makers.

Outcomes	Risk of bias	Inconsistency	Indirectness	Imprecision	Publication bias	Quality of evidence
HOMA-IR	No serious limitation	Serious limitation	No serious limitation	Serious limitation	No serious limitation	⨁⨁◯◯ Low
QUICKI	No serious limitation	Very serious limitation	No serious limitation	No serious limitation	Serious limitation	⨁◯◯◯ Very low
INS (μU/ml)	No serious limitation	Serious limitation	No serious limitation	Serious limitation	No serious limitation	⨁⨁◯◯ Low
FBG (mg/dl)	No serious limitation	No serious limitation	No serious limitation	Serious limitation	No serious limitation	⨁⨁⨁◯ Moderate
HbA1c (%)	No serious limitation	No serious limitation	No serious limitation	Very serious limitation	Serious limitation	⨁◯◯◯ Very low

HOMA-IR, homeostatic model assessment of insulin resistance; QUICKI, quantitative insulin sensitivity check index; INS, insulin; FBG, fasting blood glucose; HbA1c, glycated hemoglobin A1c.

GRADE Working Group grades of evidence.

High quality: Further research is very unlikely to change our confidence in the estimate of effect.

Moderate quality: Further research is likely to have an important impact on our confidence in the estimate of effect and may change the estimate.

Low quality: Further research is very likely to have an important impact on our confidence in the estimate of effect and is likely to change the estimate.

Very low quality: We are very uncertain about the estimate.

## 4 Discussion

We conducted a comprehensive systematic review and meta-analysis to evaluate the effects of hesperidin supplementation on insulin resistance and sensitivity, encompassing five outcome measures: HOMA-IR, QUICKI, INS, FBG, and HbA1c. Conventional meta-analysis revealed that hesperidin supplementation was associated with significant reductions in HOMA-IR and improvements in QUICKI, whereas no significant effects were observed for FBG or HbA1c. Although the overall effect on INS was non-significant, favorable outcomes were noted in specific subgroups. However, TSA results for HOMA-IR demonstrated that the cumulative Z-curve failed to cross either the conventional or sequential monitoring significance boundaries while having already traversed the RIS. This substantiates that the existing evidence confirms the absence of a significant effect of hesperidin on improving IR and portends that future analogous studies are unlikely to reverse the current directional conclusions. Although this study systematically examined potential modifying factors including formulation type, dosage, intervention duration, and population characteristics, any discussion of hesperidin’s potential value and future research directions must be undertaken with due caution within the overarching framework of “overall evidence indicating inefficacy.”

Insulin is a polypeptide hormone secreted by pancreatic β-cells that mediates multiple biological processes through binding to insulin receptor tyrosine kinase, playing a critical role in regulating glucose homeostasis, metabolism, and cellular growth (51). Previous studies have demonstrated that insulin resistance is closely associated with chronic low-grade inflammation, oxidative stress, and impaired insulin signaling pathway (52, 53). Preclinical studies have provided mechanistic insights into the actions of hesperidin: in cellular models, hesperidin inhibits the production of inflammatory cytokines in a dose-dependent manner, upregulates the expression of adiponectin and peroxisome proliferator-activated receptor gamma (54), and restores inflammation-mediated reductions in insulin receptor substrate-1 protein expression, thereby positively modulating insulin receptor signaling (21, 55). Animal studies have also confirmed that hesperidin modulates the activity of key enzymes involved in glucose metabolism (56, 57) and enhances the expression of antioxidant enzymes through activation of the nuclear factor erythroid 2-related factor 2 pathway (58, 59). Additionally, hesperidin may directly scavenge free radicals to mitigate oxidative stress-induced damage to insulin receptor substrate (60) or suppress inflammatory responses through inhibition of the nuclear factor kappa-light-chain-enhancer of activated B cells pathway (61). These studies suggest that hesperidin may improve insulin sensitivity through anti-inflammatory, antioxidant, and insulin signaling pathway-modulatory mechanisms. Although these experimental findings provide important references for understanding the potential mechanisms of hesperidin action, the translational effects in humans require further investigation.

Despite the conventional meta-analysis demonstrating a statistically significant reduction in HOMA-IR with hesperidin supplementation, this finding warrants circumspect interpretation in light of the more rigorous TSA results. The TSA indicates that the existing evidence is adequate yet insufficient to support a significant effect of hesperidin on ameliorating IR. The modest effect size and its statistical significance observed in conventional analysis likely originate from random error, amplified in the absence of correction for repeated testing. Within this overarching conclusion, trends observed in subgroup analyses should be regarded as exploratory signals and interpreted with appropriate caution. Greater reductions in HOMA-IR were observed with intervention doses exceeding 500 mg/d, prolonged treatment durations, utilization of purified formulations, and in populations with metabolic disorders or obesity (baseline BMI ≥ 30); however, these findings should be considered hypothesis-generating rather than confirmatory of efficacy. They may reflect chance occurrences influenced by methodological biases (e.g., more pronounced effects in studies with high risk of bias) or potent confounding factors. For instance, groups receiving concurrent lifestyle interventions exhibited more substantial effects. Although numerous prior meta-analyses have established the efficacy of exercise in improving metabolic parameters, particularly IR (62–64), a recent study Khalafi et al. (65) further demonstrated that exercise training combined with dietary intervention can reverse IR through multiple pathways, including enhancement of mitochondrial function, promotion of lipid metabolism, and attenuation of inflammation (66–69). Sensitivity analysis revealed that upon exclusion of all studies incorporating lifestyle interventions, the pooled effect size diminished and no longer attained statistical significance. This suggests that the observed significant benefits may be predominantly attributable to lifestyle interventions per se or their synergistic interaction, rather than the independent effect of hesperidin. Lifestyle intervention itself constitutes a potent confounding factor capable of amplifying or obscuring the true effect of hesperidin.

Contemporary evidence indicates that HOMA-IR serves as a robust predictor of future metabolic and cardiovascular events. A systematic review encompassing over 210,000 individuals confirmed that elevated HOMA-IR values are significantly associated with increased risks of T2DM (87% increased risk), hypertension (35% increased risk), and non-fatal major adverse cardiovascular events (46% increased risk) (70). Although mechanistically, the antioxidant and anti-inflammatory properties of hesperidin could theoretically confer greater benefits to metabolically compromised populations characterized by oxidative stress and chronic inflammation, and modest improvements in HOMA-IR may possess potential clinical prognostic value, the apex-level evidence assessment provided by TSA takes precedence over such indirect inferences and exploratory analyses. In summary, constrained by the existing evidence—particularly the TSA conclusions—a definitive assertion that hesperidin effectively improves IR (as measured by HOMA-IR) cannot be supported. Consequently, insufficient evidence currently exists to recommend hesperidin as a routine or adjunctive intervention for healthy individuals or those at elevated metabolic risk. Should future research endeavor to pursue this line of inquiry, investigators must acknowledge the limitations inherent in the current evidence base and commit to conducting rigorously designed, large-scale clinical trials capable of distinctly disentangling confounding factors and determining whether breakthrough effects might emerge under novel paradigms.

The present study revealed substantial heterogeneity in the effect of hesperidin on QUICKI (*I*^2^ = 97.4%, *p* < 0.001), indicating considerable variability among included studies and rendering the clinical significance of the pooled effect size uncertain, thus warranting cautious interpretation. Through subgroup analyses, we identified several factors that may influence effect sizes and heterogeneity, including study design, participants’ health status, baseline BMI, intervention dosage, intervention duration, and concurrent lifestyle modifications. Notably, among participants without metabolic disorders and with BMI < 30 kg/m^2^, hesperidin demonstrated an improvement in QUICKI (WMD: 0.19) with reduced heterogeneity (*I*^2^ = 0%). QUICKI primarily reflects whole-body insulin sensitivity; healthy individuals, compared with those with metabolic disorders maintain insulin sensitivity within normal ranges and exhibit higher baseline QUICKI values. Hesperidin may enhance QUICKI by promoting glucose transporter type 4 translocation, increasing peripheral muscle glucose uptake, and improving overall insulin sensitivity (71). Because insulin signaling pathways remain relatively intact in healthy individuals, such peripheral improvements may be more readily detectable in this population. Moreover, improvements in QUICKI were also observed among individuals with metabolic disorders and BMI ≥ 30 kg/m^2^, suggesting that hesperidin may possess regulatory potential for insulin sensitivity in these populations as well. Heterogeneity was reduced in study groups with higher doses, longer treatment durations, and concurrent lifestyle modification. These findings suggest that hesperidin supplementation may improve insulin sensitivity, although the effects are influenced by study design, baseline population characteristics, and lifestyle factors. Given the limited number of included studies and the extremely high inter-study heterogeneity, these pooled results should be interpreted with considerable caution.

Consistent with previous meta-analyses, our study did not identify a significant overall effect of hesperidin on INS. However, subgroup analyses revealed that hesperidin exhibited “dose-time-dependent” and “population-specific” patterns in improving INS levels. Supplementation with high-dose, long-duration purified formulations may exert beneficial effects on INS in individuals with metabolic disorders and obesity. These findings are consistent with the subgroup analysis results showing reduced HOMA-IR and elevated QUICKI, suggesting that hesperidin’s mechanism of action may involve improving peripheral insulin sensitivity rather than directly stimulating insulin secretion or substantially reducing circulating insulin levels. Notably, although subgroup analyses demonstrated significant improvements in INS and HOMA-IR under specific conditions, the overall meta-analysis did not achieve statistical significance for INS or FBG. This indicates that hesperidin’s effects may be condition-dependent rather than universally applicable. Furthermore, as multiple subgroup comparisons were conducted without statistical correction, there exists a risk of false-positive findings; therefore, these subgroup results should be regarded as exploratory. Additionally, sensitivity analysis demonstrated that INS results were sensitive to certain studies, and the GRADE assessment rated the quality of evidence for INS as low, suggesting that subgroup effects may be influenced by methodological biases that overestimate the true effect.

The FBG analysis revealed no significant beneficial effect of hesperidin supplementation on FBG, regardless of intervention type, dosage, duration, or population characteristics. This finding contradicts the 2023 meta-analysis by Huang et al. (26), which reported that purified hesperidin supplementation improved FBG, with significant reductions observed in groups receiving >500 mg/day for >6 weeks. This discrepancy may be attributable to the fact that the previous study exclusively included oral purified hesperidin, whereas the present analysis incorporated studies using hesperidin compounds, potentially resulting in a dilution effect. In orange juice trials, control groups typically also consumed low doses of hesperidin, which may have narrowed between-group differences and led to underestimation of effect sizes, biasing results toward the null. Furthermore, hesperidin is absorbed in the gastrointestinal tract, particularly in the colon, where gut microbiota convert it to its aglycone form (hesperetin) (72). Hesperetin is subsequently absorbed through intestinal epithelial cells and released into the bloodstream as glucuronide and sulfate conjugates (73). Consequently, the bioavailability of hesperidin is influenced by the form of intake, the molecular structure of the compound, and host intrinsic characteristics (gut microbiota) (74). Additionally, hesperidin may need to achieve effective local tissue concentrations to activate AMP-activated protein kinase /insulin signaling pathways; insufficient dosage or duration may be inadequate to influence serum FBG levels, and higher doses or longer treatment durations may be required to observe effects on FBG. These factors may collectively explain the lack of significant improvement in FBG observed in the present study. Integrating the TSA conclusion of “futility” for HOMA-IR, this study provides no evidence supporting a significant direct hypoglycemic effect of hesperidin, nor an indirect glucose-lowering effect mediated through amelioration of IR.

Our analysis also revealed a significant non-linear dose–response relationship between hesperidin dosage and QUICKI improvement, demonstrating an ascending trend within the range of 700–1,000 mg daily, with maximal effect observed at 1,000 mg/d. This suggests that hesperidin may exert its QUICKI-enhancing effects across a broad dosage spectrum. Additionally, intervention duration exhibited a significant non-linear relationship with improvements in QUICKI and INS, whereby beneficial effects became more pronounced over time before reaching a plateau, indicating that hesperidin supplementation for 10–12 weeks may represent the optimal timeframe for therapeutic efficacy. However, these curve-fitting analyses relied on pooled data characterized by limited sample sizes and substantial inter-study heterogeneity, resulting in unstable model estimates. Consequently, the clinical reproducibility and generalizability of the observed U-shaped or inverted U-shaped curves remain questionable, necessitating extreme caution in the interpretation of these findings.

A notable finding of the present study pertains to the influence of methodological quality on effect estimates. When interpreting the results, we thoroughly considered the risk of bias in the included studies. Notably, significant improvements in HOMA-IR and INS were predominantly observed in studies with higher risk of bias, potentially indicating that these results were influenced to some extent by systematic errors, thereby overestimating the true effect sizes. However, upon further analysis, we determined that the elevated risk of bias primarily stemmed from methodological issues such as inadequate reporting of allocation concealment—factors that typically exert minimal impact on objective indicators such as HOMA-IR and INS. Furthermore, sensitivity analyses demonstrated that effect estimates remained close to statistical significance following the exclusion of high-risk-of-bias studies, suggesting that the influence of bias risk on the robustness of core outcomes may be limited. Conversely, improvements in QUICKI were also evident in studies with low risk of bias, which strengthens our confidence in these findings. Nevertheless, given the presence of extremely high heterogeneity, these results warrant cautious interpretation.

The present study possesses several strengths. First, the study design and reporting were conducted with reference to and in accordance with systematic review standards such as AMSTAR-2, thereby enhancing methodological rigor. Second, we implemented stringent inclusion and exclusion criteria and restricted our analysis exclusively to RCTs to ensure the incorporation of high-quality data in the pooled analysis. Third, the included trials demonstrated broad geographic distribution, encompassing regions such as Europe, the Middle East, South America, and Africa, which enhances the generalizability of our conclusions. Fourth, this study incorporated both purified hesperidin supplements and hesperidin-rich composite formulations, enabling comprehensive evaluation of their effects across diverse application contexts. Consequently, this meta-analysis represents the most current, rigorous, comprehensive, and in-depth investigation to date, serving to redefine and reinforce prior research conclusions. Fifth, we conducted subgroup analyses stratified by study type, intervention type, dosage and duration, baseline BMI, participants’ health status, concomitant lifestyle modification, and risk of bias to examine the intervention effects of hesperidin across various factors. Additionally, sensitivity analyses were performed to accurately identify the primary sources of heterogeneity. Furthermore, we employed the GRADE framework to assess the overall quality of clinical evidence and the strength of recommendations for each outcome.

Nevertheless, this study is subject to several limitations. First, as none of the included primary studies measured hesperidin bioavailability—which is influenced by factors such as intake form, gut microbiota composition, and molecular structure—establishing a definitive relationship between hesperidin concentration and improvement in insulin resistance remains challenging. Second, the quality of evidence for primary outcome measures is limited: although QUICKI demonstrated statistical significance, it was characterized by substantial heterogeneity and evidence of publication bias; results for HbA1c remain inconclusive due to the paucity of included studies. Third, methodological challenges exist: risk of bias assessment classified half of the studies as harboring “some concerns,” implying that the majority of studies may compromise confidence in the estimated effects; although extensive subgroup analyses were conducted to explore sources of heterogeneity, the absence of correction for multiple comparisons inevitably elevates the risk of false-positive findings; furthermore, statistical parameters in certain studies relied on imputation, potentially introducing measurement error. Fourth, the existing evidence derives predominantly from trials conducted in populations with specific metabolic disorders, and generalizability to broader populations remains unverified. Fifth, lifestyle modification were implemented concurrently with hesperidin supplementation in numerous studies, constraining our ability to infer the independent causal effect of hesperidin from pooled data. Sixth, although TSA results indicate “sufficient evidence,” the calculations are predicated upon currently included RCTs; despite TSA being designed to control for random error, clinical heterogeneity among included studies may exert some influence on RIS estimation and ultimate conclusions. Additionally, several studies received industry funding, and although most declared that sponsors were not involved in study conduct, the possibility of funding bias cannot be entirely excluded. Finally, GRADE assessment indicated that evidence quality for most outcomes was “low” or “very low,” primarily attributable to heterogeneity and risk of bias. Consequently, current evidence remains uncertain, and our effect estimates may be subject to bias. Future large-scale, long-term RCTs with rigorous designs, independent funding sources, and bioavailability measurements are warranted to furnish more definitive evidence.

## 5 Conclusion

Based on current conventional meta-analysis results, hesperidin supplementation may contribute to improvements in HOMA-IR and QUICKI; however, no overall significant effects were observed for INS, FBG, or HbA1c. Higher-level evidence provided by TSA indicates that hesperidin confers no definitive therapeutic efficacy in reducing IR, and this conclusion is unlikely to be altered even with the accrual of additional studies. The true effect of hesperidin may be modulated by factors including formulation type, dosage, intervention duration, and population characteristics; nonetheless, current evidence does not support its use as an effective intervention for improving IR or glycemic control. Future research may focus on distinct subgroups or mechanistic exploration; however, based on the present analysis, the clinical utility of hesperidin for treating insulin resistance appears limited.

## Data Availability

The original contributions presented in the study are included in the article/Supplementary material, further inquiries can be directed to the corresponding author.

## Glossary

BMI: Body mass index
CI: Confidence interval
DM: Diabetes mellitus
FBG: Fasting blood glucose
GRADE: Grading of recommendations, assessment, development and evaluation
HOMA-IR: Homeostatic model assessment of insulin resistance
HbA1c: Glycated hemoglobin A1c
I^2^: I-square
INS: Insulin
IR: Insulin resistance
MetS: Metabolic syndrome
NAFLD: Non-alcoholic fatty liver disease
QUICKI: Quantitative insulin sensitivity check index
RCTs: Randomized controlled trials
RoB 2: Cochrane risk of bias 2
RIS: Required information size
SEM: Standard error of the mean
SD: Standard deviation
T2DM: Type 2 diabetes mellitus
TSA: Trial sequential analysis
WMD: Weighted mean difference
