# Supplementary material for: The effects of hesperidin supplementation on insulin resistance and sensitivity in adults: a systematic review and meta-analysis of randomized controlled trials
Source: Front Nutr. 2026 Jan 9;12:1724786. doi: 10.3389/fnut.2025.1724786 (PMC12827108; doi:10.3389/fnut.2025.1724786)
Supplement: Supplementary file 1 [file Table_1.docx]

Supplementary Table 1. The search strategy used in all databases.

| **Database** | **Search strategy** |
| --- | --- |
| **PubMed** | (((((((Hesperidin[MeSH Terms]) OR (hesperitin[Title/Abstract])) OR (citrus flavonoid[Title/Abstract])) OR (orange juice[Title/Abstract])) OR (orange polyphenols[Title/Abstract])) OR (citrus flavanones[Title/Abstract])) AND (((((((((((((((((((((((((Insulin Resistance[MeSH Terms]) OR (Insulin[Title/Abstract])) OR (blood insulin[Title/Abstract])) OR (fasting blood insulin[Title/Abstract])) OR (FBI[Title/Abstract])) OR (insulin sensitivity[Title/Abstract])) OR (homeostatic model assessment of insulin resistance[Title/Abstract])) OR (HOMA-IR[Title/Abstract])) OR (Matsuda index[Title/Abstract])) OR (quantitative insulin sensitivity check index[Title/Abstract])) OR (QUICKI[Title/Abstract])) OR (Bennett ISI[Title/Abstract])) OR (Glucose[Title/Abstract])) OR (glycemic[Title/Abstract])) OR (glycemic control[Title/Abstract])) OR (glucose control[Title/Abstract])) OR (glycemic indices[Title/Abstract])) OR (blood glucose[Title/Abstract])) OR (blood sugar[Title/Abstract])) OR (fast plasma glucose[Title/Abstract])) OR (FPG[Title/Abstract])) OR (fasting blood sugar[Title/Abstract])) OR (FBS[Title/Abstract])) OR (glycated hemoglobin[Title/Abstract])) OR (HbA1c[Title/Abstract])))) AND (((((((randomized controlled trial[Publication Type]) OR (randomized[Title/Abstract])) OR (placebo[Title/Abstract])) OR (random[Title/Abstract])) OR (clinical trial[Title/Abstract])) OR (trial[Title/Abstract])) OR (RCT[Title/Abstract])) |
| **Scopus** | ("Hesperidin" OR "hesperitin" OR "citrus flavonoid" OR "orange juice" OR "orange polyphenols" OR "citrus flavanones") AND ("Insulin Resistance" OR "Insulin" OR "blood insulin" OR "fasting blood insulin" OR "FBI" OR "insulin sensitivity" OR "homeostatic model assessment of insulin resistance" OR "HOMA-IR" OR "Matsuda index" OR "QUICKI" OR "quantitative insulin sensitivity check index" OR "Bennett ISI" OR "glucose" OR "glycemic" OR "glycemic control" OR "glucose control" OR "glycemic indices" OR "blood glucose" OR "blood sugar" OR "fast plasma glucose" OR "FPG" OR "fasting blood sugar" OR "FBS" OR "glycated hemoglobin" OR "HbA1c") AND ("randomized controlled trial" OR "randomized" OR "placebo" OR "random" OR "clinical trial" OR "trial" OR "RCT") |
| **Embase** | ('Hesperidin':ab,ti OR 'hesperitin':ab,ti OR 'citrus flavonoid':ab,ti OR 'orange juice':ab,ti OR 'orange polyphenols':ab,ti OR 'citrus flavanones':ab,ti) AND ('Insulin Resistance':ab,ti OR 'Insulin':ab,ti OR 'blood insulin':ab,ti OR 'fasting blood insulin':ab,ti OR 'FBI':ab,ti OR 'insulin sensitivity':ab,ti OR 'homeostatic model assessment of insulin resistance':ab,ti OR 'HOMA-IR':ab,ti OR 'Matsuda index':ab,ti OR 'QUICKI':ab,ti OR 'quantitative insulin sensitivity check index':ab,ti OR 'Bennett ISI':ab,ti OR 'glucose':ab,ti OR 'glycemic':ab,ti OR 'glycemic control':ab,ti OR 'glucose control':ab,ti OR 'glycemic indices':ab,ti OR 'blood glucose':ab,ti OR 'blood sugar':ab,ti OR 'fast plasma glucose':ab,ti OR 'FPG':ab,ti OR 'fasting blood sugar':ab,ti OR 'FBS':ab,ti OR 'glycated hemoglobin':ab,ti OR 'HbA1c':ab,ti) AND ('randomized controlled trial':ab,ti OR 'randomized':ab,ti OR 'placebo':ab,ti OR 'random':ab,ti OR 'clinical trial':ab,ti OR 'trial':ab,ti OR 'RCT':ab,ti) |
| **Web of science** | TS=(Hesperidin OR hesperitin OR citrus flavonoid OR orange juice OR orange polyphenols OR citrus flavanones) AND TS=(Insulin Resistance OR Insulin OR blood insulin OR fasting blood insulin ORFBI OR insulin sensitivity OR homeostatic model assessment of insulin resistance OR HOMA-IR OR Matsuda index OR QUICKI OR quantitative insulin sensitivity check index OR Bennett ISI OR glucose OR glycemic OR glycemic control OR glucose control OR glycemic indices OR blood glucose OR blood sugar OR fast plasma glucose OR FPG OR fasting blood sugar OR FBS OR glycated hemoglobin OR HbA1c) AND TS=( randomized controlled trial OR randomized OR placebo OR random OR clinical trial OR trial OR RCT) |
| **Cochrane library** | (Hesperidin OR hesperitin OR citrus flavonoid OR orange juice OR orange polyphenols OR citrus flavanones):ti,ab,kw AND (Insulin Resistance OR Insulin OR blood insulin OR fasting blood insulin OR FBI OR insulin sensitivity OR homeostatic model assessment of insulin resistance OR HOMA-IR OR Matsuda index OR QUICKI OR quantitative insulin sensitivity check index OR Bennett ISI OR glucose OR glycemic OR glycemic control OR glucose control OR glycemic indices OR blood glucose OR blood sugar OR fast plasma glucose OR FPG OR fasting blood sugar OR FBS OR glycated hemoglobin OR HbA1c):ti,ab,kw |

Supplementary Table 2. Excluded Trials and Reasons for Exclusion.

| **Excluded trials** | **Reason for exclusion** |
| --- | --- |
| Ekhlasi 2015(1) | This randomized trial did not report outcomes of interest, and the hesperidin content of the orange juice was not available. |
| Buscemi 2012(2) | This randomized trial did not report the outcomes of interest. |
| Li 2017(3) | This randomized trial did not report the outcomes of interest. |
| Rangel-Huerta 2017(4) | This randomized trial did not report the outcomes of interest. |
| Li 2020(5) | This randomized trial did not report the outcomes of interest. |
| Enderle 2024(6) | This randomized trial did not report the outcomes of interest and is merely a conference abstract. |
| Martinez-Noguera 2022(7) | This randomized trial did not report the outcomes of interest. |
| Unretrievable records identified only by trial number (NCT01201603)(8) | Only the trial registration number for this study was identified; the full text could not be accessed. |
| Unretrievable records identified only by trial number (NCT01773486)(9) | Only the trial registration number for this study was identified; the full text could not be accessed. |
| Constans 2015(10) | This randomized controlled trial provided insufficient data reporting. |
| Unretrievable records identified only by trial number (NCT02610491)(11) | Only the trial registration number for this study was identified; the full text could not be accessed. |
| Unretrievable records identified only by trial number (ISRCTN11708820)(12) | Only the trial registration number for this study was identified; the full text could not be accessed. |
| Unretrievable records identified only by trial number (ISRCTN15062713)(13) | Only the trial registration number for this study was identified; the full text could not be accessed. |
| Price 2021(14) | This randomized trial did not report the hesperidin content of the orange juice. |
| Santos 2022(15) | The control group in this clinical trial failed to meet the inclusion criteria, and the outcome measures lacked sufficient integrity. |
| Santana 2022(16) | The control group in this clinical trial failed to meet the inclusion criteria, and the outcome measures lacked sufficient integrity. |
| Notarnicola 2024(17) | This clinical trial intervention measure does not meet the inclusion criteria and the dosage of hesperidin has not been clearly reported. |
| Eghtesadi 2017(18) | This is a conference abstract. |
| Cesar 2020(19) | This is a conference abstract. |
| Büsing 2019(20) | This clinical trial was a quasi-randomized controlled cross-over study and was conducted over a short duration (2 weeks). |

**References**

1. Ekhlasi G, Shidfar F, Agah S, Merat S, Hosseini AF. Effects of pomegranate and orange juice on antioxidant status in non-alcoholic fatty liver disease patients: a randomized clinical trial. International journal for vitamin and nutrition research. 2015;85(5-6):292-8.

2. Buscemi S, Rosafio G, Arcoleo G, Mattina A, Canino B, Montana M, et al. Effects of red orange juice intake on endothelial function and inflammatory markers in adult subjects with increased cardiovascular risk. Am J Clin Nutr. 2012;95(5):1089-95.

3. Li L. Effects of citrus flavonoids on endothelial function and cardiovascular health: University of Leeds; 2017.

4. Rangel-Huerta OD, Aguilera CM, Perez-de-la-Cruz A, Vallejo F, Tomas-Barberan F, Gil A, et al. A serum metabolomics-driven approach predicts orange juice consumption and its impact on oxidative stress and inflammation in subjects from the BIONAOS study. Mol Nutr Food Res. 2017;61(2).

5. Li L, Lyall GK, Martinez-Blazquez JA, Vallejo F, F AT-B, Birch KM, et al. Blood Orange Juice Consumption Increases Flow-Mediated Dilation in Adults with Overweight and Obesity: A Randomized Controlled Trial. J Nutr. 2020;150(9):2287-94.

6. Enderle J, Dörner R, Gilcher C, Steingaß C, Schweiggert R, Müller MJ, et al. Einfluss von Vitamin C und Hesperidin auf die Harnsäurekonzentration im Blut und Urin bei gesunden Personen. Aktuelle Ernährungsmedizin. 2024;49(03):P28.

7. Martínez-Noguera FJ, Alcaraz PE, Carlos-Vivas J, Marín-Pagán C. Chronic Supplementation of 2S-Hesperidin Improves Acid-Base Status and Decreases Lactate at FatMax, at Ventilatory Threshold 1 and 2 and after an Incremental Test in Amateur Cyclists. Biology (Basel). 2022;11(5).

8. The Effects of Orange Juice on Insulin Sensitivity and Plasma Lipids [Internet]. 2010. Available from: <https://clinicaltrials.gov/study/NCT01201603>.

9. An Exploratory Study to Evaluate the Ability of the Citrus Polyphenol Hesperidin to Improve Insulin Sensitivity in Healthy Subjects and to Ameliorate Insulin Resistance in Obese Subjects [Internet]. 2013. Available from: <https://clinicaltrials.gov/study/NCT01773486>.

10. Constans J, Bennetau-Pelissero C, Martin JF, Rock E, Mazur A, Bedel A, et al. Marked antioxidant effect of orange juice intake and its phytomicronutrients in a preliminary randomized cross-over trial on mild hypercholesterolemic men. Clin Nutr. 2015;34(6):1093-100.

11. The Effect of Hesperidin Administration on Glucose / Insulin Metabolism [Internet]. 2015. Available from: <https://clinicaltrials.gov/study/NCT02610491>.

12. Isrctn. The beneficial effect on cardiovascular function of a food supplement based on a mixture of Mentha spicata L. extract, Amaranthus caudatus L. seed flour, flavonoids and vitamins. <https://trialsearchwhoint/Trial2aspx?TrialID=ISRCTN11708820>. 2024.

13. Isrctn. Study of the efficacy of a food supplement based on extracts of lemon and orange, hesperidin and chromium for the maintenance of normal carbohydrate metabolism. <https://trialsearchwhoint/Trial2aspx?TrialID=ISRCTN15062713>. 2025.

14. Price CA, Medici V, Nunez MV, Lee V, Sigala DM, Benyam Y, et al. A Pilot Study Comparing the Effects of Consuming 100% Orange Juice or Sucrose-Sweetened Beverage on Risk Factors for Cardiometabolic Disease in Women. Nutrients. 2021;13(3).

15. Santos KGD, Yoshinaga MY, Glezer I, Chaves-Filho AB, Santana AA, Kovacs C, et al. Orange juice intake by obese and insulin-resistant subjects lowers specific plasma triglycerides: A randomized clinical trial. Clin Nutr ESPEN. 2022;51:336-44.

16. de Santana AA, de Castro Tobaruela E, dos Santos KG, Sparvoli LG, do Amaral CK, Magnoni CD, et al. ‘Pera’orange and ‘Moro’blood orange juice improves oxidative stress and inflammatory response biomarkers and modulates the gut microbiota of individuals with insulin resistance and different obesity classes. Obesities. 2022;2(4):389-412.

17. Notarnicola M, Tutino V, De Nunzio V, Cisternino AM, Cofano M, Donghia R, et al. Daily Orange Consumption Reduces Hepatic Steatosis Prevalence in Patients with Metabolic Dysfunction-Associated Steatotic Liver Disease: Exploratory Outcomes of a Randomized Clinical Trial. Nutrients. 2024;16(18).

18. Eghtesadi S, Mohammadi M, Vafa M, Heidari I, Salehi M, Khadem HH, et al., editors. Effects of hesperidin supplementation on glycemic control, lipid profile and inflammatory factors in patients with type 2 diabetes: A randomized, double-blind and placebo-controlled clinical trial. Endocrine Abstracts; 2016: Bioscientifica.

19. Cesar T, Benassi R, Ponce O, Nasser M. Orange juice combined to a healthy-eating pattern improved endothelial function and reduced global risk of CHD in metabolic syndrome patients. Proceedings of the Nutrition Society. 2020;79(OCE2):E241.

20. Büsing F, Hägele FA, Nas A, Döbert LV, Fricker A, Dörner E, et al. High intake of orange juice and cola differently affects metabolic risk in healthy subjects. Clin Nutr. 2019;38(2):812-9.

Supplementary Table 3. Subgroup analysis of the effects of hesperidin supplementation on the HOMA-IR in adults.

|  |  |  |  | **Heterogeneity** | | |
| --- | --- | --- | --- | --- | --- | --- |
|  | **NO.** | **WMD^1^ (95%CI)** | ***P*-value** | ***P*_heterogeneity_** | **I^2^** | ***P* _between sub-groups_** |
| **Overall effect** | 10 | -0.43 (-0.82, -0.03) | **0.034** | 0.044 | 48.0% |  |
| **Research type** |  |  |  |  |  |  |
| Parallel trial | 9 | -0.61 (-0.92, -0.30) | **＜0.001** | 0.302 | 15.8% | **0.005** |
| Crossover trial | 1 | -0.30 (-0.25, 0.85) | 0.289 | ＜0.001 | 0.0% |  |
| **Intervention types** |  |  |  |  |  |  |
| Purified hesperidin | 5 | -0.89 (-1.28, -0.50) | **＜0.001** | 0.490 | 0.0% | **0.001** |
| Hesperidin complexes | 5 | 0.07 (-0.30, 0.45) | 0.699 | 0.761 | 0.0% |  |
| **Intervention dose (mg/d)** |  |  |  |  |  |  |
| ≤ 500 | 5 | -0.11 (-0.59, 0.37) | 0.647 | 0.953 | 0.0% | 0.170 |
| ＞500 | 5 | -0.52 (-0.85, -0.19) | **0.002** | 0.005 | 72.9% |  |
| **Trial duration (week)** |  |  |  |  |  |  |
| ≤ 6 | 2 | 0.13 (-0.71, 0.98) | 0.759 | 0.717 | 0.0% | 0.202 |
| ＞6 | 8 | -0.45 (-0.74, -0.16) | **0.002** | 0.029 | 55% |  |
| **Health status** |  |  |  |  |  |  |
| No metabolic disorders | 1 | -0.22 (-1.08, 0.64) | 0.616 | ＜0.001 | 0.0% | 0.683 |
| Metabolic disorders | 9 | -0.41 (-0.69, -0.12) | **0.005** | 0.029 | 53.4% |  |
| **Baseline BMI^1^ (kg/m^2^)** |  |  |  |  |  |  |
| ＜30 | 2 | -0.17 (-0.90, 0.55) | 0.639 | 0.844 | 0.0% | 0.527 |
| ≥ 30 | 8 | -0.43 (-0.72, -0.13) | **0.004** | 0.018 | 58.5% |  |
| **Lifestyle modification** |  |  |  |  |  |  |
| NO | 3 | 0.12 (-0.32, 0.56) | 0.578 | 0.584 | 0.0% | **0.004** |
| Yes | 7 | -0.70 (-1.05, -0.36) | **＜0.001** | 0.255 | 22.9% |  |
| **Study quality** |  |  |  |  |  |  |
| Low risk group | 4 | -0.17 (-0.67, 0.33) | 0.510 | 0.803 | 0.0% | 0.298 |
| Moderate to high risk group | 6 | -0.48 (-0.81, -0.16) | **0.003** | 0.009 | 67.2% |  |

WMD^1:^ weighted mean difference; BMI: body mass index.

Supplementary Figure1. Trial sequential analysis of the effect of hesperidin supplementation on HOMA-IR.

**
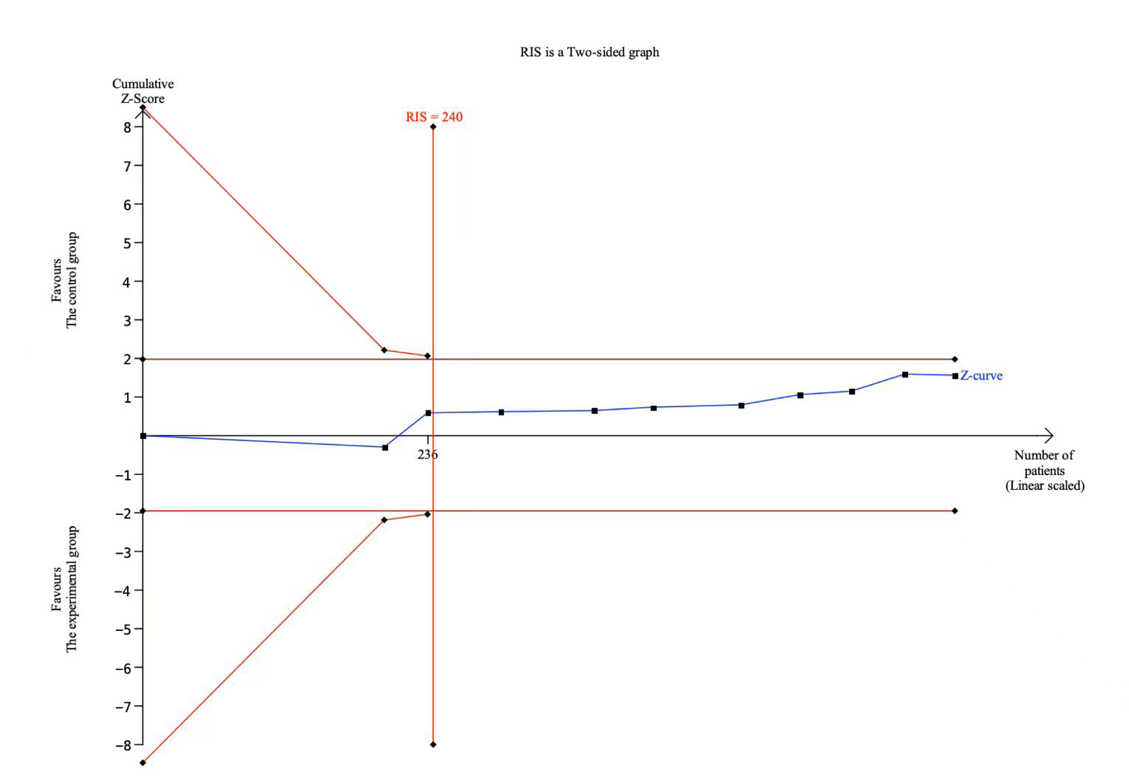
**

The blue line (Z-curve) represents the cumulative Z‑score, with each point indicating the result of an individual trial. The vertical red line on the right denotes the required information size (RIS). The horizontal red lines indicate the conventional two‑sided statistical significance boundaries (P = 0.05) for benefit (above the central black line) and harm (below the central black line), respectively. The outer curved red lines represent the trial sequential monitoring boundaries for benefit (below) and harm (above). The inner wedge formed by the red lines above and below the central black line represents the futility boundaries.

Supplementary Table 4. Subgroup analysis of the effects of hesperidin supplementation on the QUICKI in adults.

|  |  |  | **Heterogeneity** | | | |
| --- | --- | --- | --- | --- | --- | --- |
|  | **NO.** | **WMD^1^ (95%CI)** | ***P*-value** | ***P*_heterogeneity_** | **I^2^** | ***P* _between sub-groups_** |
| **Overall effect** | 5 | 0.05 (0.01, 0.08) | **0.005** | ＜0.001 | 97.4% |  |
| **Research type** |  |  |  |  |  |  |
| Parallel trial | 4 | 0.03 (0.03, 0.04) | **＜0.001** | ＜0.001 | 97.5% | **＜0.001** |
| Crossover trial | 1 | 0.00 (-001, 0.01) | 1.000 | ＜0.001 | 0.0% |  |
| **Intervention dose (mg/d)** |  |  |  |  |  |  |
| ≤ 500 | 2 | 0.02 (0.01, 0.04) | **＜0.001** | ＜0.001 | 99.3% | 0.190 |
| ＞500 | 3 | 0.02 (0.02, 0.03) | **＜0.001** | 0.376 | 0.0% |  |
| **Trial duration (week)** |  |  |  |  |  |  |
| ≤ 6 | 2 | 0.02 (0.01, 0.04) | **＜0.001** | ＜0.001 | 99.3% | 0.190 |
| ＞6 | 3 | 0.02 (0.02, 0.03) | **＜0.001** | 0.376 | 0.0% |  |
| **Health status** |  |  |  |  |  |  |
| No metabolic disorders | 1 | 0.19 (0.16, 0.22) | **＜0.001** | ＜0.001 | 0.0% | **＜0.001** |
| Metabolic disorders | 4 | 0.02 (0.01, 0.02) | **＜0.001** | ＜0.001 | 84.2% |  |
| **Baseline BMI^1^ (kg/m^2^)** |  |  |  |  |  |  |
| ＜30 | 1 | 0.19 (0.16, 0.22) | **＜0.001** | ＜0.001 | 0.0% | **＜0.001** |
| ≥ 30 | 4 | 0.02 (0.01, 0.02) | **＜0.001** | ＜0.001 | 84.2% |  |
| **Lifestyle modification** |  |  |  |  |  |  |
| NO | 2 | 0.02 (0.01, 0.02) | **＜0.001** | ＜0.001 | 99.3% | 0.190 |
| Yes | 3 | 0.02 (0.02, 0.03) | **＜0.001** | 0.376 | 0.0% |  |
| **Study quality** |  |  |  |  |  |  |
| Low risk group | 2 | 0.02 (0.01, 0.02) | **＜0.001** | ＜0.001 | 99.3% | 0.190 |
| Moderate to high risk group | 3 | 0.02 (0.02, 0.03) | **＜0.001** | 0.376 | 0.0% |  |

WMD^1:^ weighted mean difference; BMI: body mass index.

Supplementary Table 5. Subgroup analysis of the effects of hesperidin supplementation on the INS in adults.

|  |  | **Heterogeneity** | | | | |
| --- | --- | --- | --- | --- | --- | --- |
|  | **NO.** | **WMD^1^ (95%CI)** | ***P*-value** | ***P*_heterogeneity_** | **I^2^** | ***P*_between sub-groups_** |
| **Overall effect** | 11 | -1.61 (-3.30, 0.08) | 0.062 | 0.011 | 56.2% |  |
| **Research type** |  |  |  |  |  |  |
| Parallel trial | 8 | -2.71 (-3.98, -1.44) | **＜0.001** | 0.151 | 34.8% | **0.001** |
| Crossover trial | 3 | 0.80 (-0.84, 2.43) | 0.339 | 0.592 | 0.0% |  |
| **Intervention types** |  |  |  |  |  |  |
| Purified hesperidin | 7 | -3.25 (-4.63, -1.88) | **＜0.001** | 0.395 | 4.1% | **＜0.001** |
| Hesperidin complexes | 4 | 0.76 (-0.71, 2.23) | 0.312 | 0.737 | 0.0% |  |
| **Intervention dose (mg/d)** |  |  |  |  |  |  |
| ≤ 500 | 6 | -0.09 (-2.14, 1.95) | 0.982 | 0.871 | 0.0% | 0.154 |
| ＞500 | 5 | -1.80 (-2.95, -0.65) | **0.002** | ＜0.001 | 78.9% |  |
| **Trial duration (week)** |  |  |  |  |  |  |
| ≤ 6 | 4 | 0.41 (-2.16, 2.97) | 0.755 | 0.703 | 0.0% | 0.136 |
| ＞6 | 7 | -1.71 (-2.80, -0.62) | **0.002** | 0.004 | 68.7% |  |
| **Health status** |  |  |  |  |  |  |
| No metabolic disorders | 2 | 0.46 (-3.94, 4.85) | 0.839 | 0.293 | 9.6% | 0.398 |
| Metabolic disorders | 9 | -1.49 (-2.52, -0.46) | **0.005** | 0.007 | 61.9% |  |
| **Baseline BMI^1^ (kg/m^2^)** |  |  |  |  |  |  |
| ＜30 | 2 | 0.46 (-3.94, 4.85) | 0.839 | 0.293 | 9.6% | 0.398 |
| ≥ 30 | 9 | -1.49 (-2.52, -0.46) | **0.005** | 0.007 | 61.9% |  |
| **Lifestyle modification** |  |  |  |  |  |  |
| NO | 4 | 0.93 (-0.67, 2.53) | 0.256 | 0.647 | 0.0% | **＜0.001** |
| Yes | 7 | -2.89 (-4.18, -1.60) | **＜0.001** | 0.244 | 24.2% |  |
| **Study quality** |  |  |  |  |  |  |
| Low risk group | 4 | -0.52 (-2.72, 1.69) | 0.646 | 0.394 | 0.0% | 0.385 |
| Moderate to high risk group | 7 | -1.62 (-2.74, -0.49) | **0.005** | 0.004 | 68.6% |  |

WMD^1:^ weighted mean difference; BMI: body mass index.

Supplementary Table 6. Subgroup analysis of the effects of hesperidin supplementation on the FBG in adults.

|  |  | **Heterogeneity** | | | | |
| --- | --- | --- | --- | --- | --- | --- |
|  | **NO.** | **WMD^1^ (95%CI)** | ***P*-value** | ***P*_heterogeneity_** | **I^2^** | ***P*_between sub-groups_** |
| **Overall effect** | 15 | -0.59 (-2.56,1.37) | 0.555 | 0.752 | 0.0% |  |
| **Baseline FBG (mg/dL)** |  |  |  |  |  |  |
| ≤ 100 | 5 | -0.45 (-3.71, 2.82) | 0.789 | 0.974 | 0.0% | 0.911 |
| ＞100 | 10 | -0.68 (-3.15, 1.79) | 0.382 | 0.382 | 6.5% |  |
| **Research types** |  |  |  |  |  |  |
| Parallel trial | 11 | -0.51 (-2.73, 1.70) | 0.648 | 0.475 | 0.0% | 0.879 |
| Crossover trial | 4 | -0.89 (-5.21, 3.42) | 0.685 | 0.920 | 0.0% |  |
| **Intervention types** |  |  |  |  |  |  |
| Purified hesperidin | 10 | -0.01 (-2.47, 2.45) | 0.994 | 0.701 | 0.0% | 0.440 |
| Hesperidin complexes | 5 | -1.62 (-2.56, 1.37) | 0.331 | 0.752 | 0.0% |  |
| **Intervention dose (mg/d)** |  |  |  |  |  |  |
| ≤ 500 | 9 | -1.34 (-4.30, 1.61) | 0.373 | 0.748 | 0.0% | 0.505 |
| ＞500 | 6 | 0.00 (-2.63, 2.64) | 0.999 | 0.467 | 0.0% |  |
| **Trial duration (week)** |  |  |  |  |  |  |
| ≤ 6 | 6 | -0.22 (-3.83, 3.39) | 0.906 | 0.976 | 0.0% | 0.808 |
| ＞6 | 9 | -0.75 (-3.10, 1.60) | 0.530 | 0.321 | 13.6% |  |
| **Health status** |  |  |  |  |  |  |
| No metabolic disorders | 3 | -0.77 (-5.21, 3.68) | 0.736 | 0.802 | 0.0% | 0.933 |
| Metabolic disorders | 12 | -0.55 (-2.75, 1.64) | 0.623 | 0.559 | 0.0% |  |
| **Baseline BMI^1^ (kg/m^2^)** |  |  |  |  |  |  |
| ＜30 | 5 | 0.72 (-2.83, 4.26) | 0.692 | 0.760 | 0.0% | 0.384 |
| ≥ 30 | 10 | -1.18 (-3.54, 1.19) | 0..330 | 0.752 | 0.0% |  |
| **Lifestyle modification** |  |  |  |  |  |  |
| NO | 8 | 0.37 (-2.48, 3.21) | 0.801 | 0.953 | 0.0% | 0.361 |
| Yes | 7 | -1.47 (-4.19, 1.25) | 0.290 | 0.305 | 16.4% |  |
| **Study quality** |  |  |  |  |  |  |
| Low risk group | 7 | 1.53 (-1.42, 4.47) | 0.310 | 0.968 | 0.0% | 0.058 |
| Moderate to high risk group | 8 | -2.30 (-4.94, 0.35) | 0.088 | 0.058 | 0.0% |  |

WMD^1:^ weighted mean difference; BMI: body mass index.

Supplementary Table 7. Results of sensitivity analyses with exclusion of the listed trials.

| **Removed trials** | **No of studies** | **WMD (95%CI)** | ***P*-value** | **I^2^** | ***P*_heterogeneity_** |
| --- | --- | --- | --- | --- | --- |
| **HOMA-IR** |  |  |  |  |  |
| **Ribeiro 2017** |  |  |  |  |  |
| Before sensitivity analysis | 10 | -0.43 (-0.82, -0.03) | 0.034 | 48.0% | 0.044 |
| After sensitivity analysis | 9 | -0.43 (-0.84, -0.01) | 0.044 | 53.7% | 0.027 |
| **INS** |  |  |  |  |  |
| **Ribeiro 2017** |  |  |  |  |  |
| Before sensitivity analysis | 11 | -1.61 (-3.30, 0.08) | 0.062 | 56.2% | 0.011 |
| After sensitivity analysis | 10 | -1.60 (-3.34, 0.15) | 0.073 | 60.5% | 0.007 |
| **Rangel-Huerta 2015** |  |  |  |  |  |
| Before sensitivity analysis | 11 | -1.61 (-3.30, 0.08) | 0.062 | 56.2% | 0.011 |
| After sensitivity analysis | 10 | -2.37 (-3.78, -0.95) | 0.001 | 21.2% | 0.248 |
| **Salden 2016** |  |  |  |  |  |
| Before sensitivity analysis | 11 | -1.61 (-3.30, 0.08) | 0.062 | 56.2% | 0.011 |
| After sensitivity analysis | 10 | -1.82 (-3.52, -0.12) | 0.036 | 57.2% | 0.013 |
| **FBG** |  |  |  |  |  |
| **Ribeiro 2017** |  |  |  |  |  |
| Before sensitivity analysis | 15 | -0.59 (-2.56, 1.37) | 0.555 | 0.0% | 0.752 |
| After sensitivity analysis | 14 | -0.59 (-2.57, 1.39) | 0.560 | 0.0% | 0.683 |
| **Ponce 2019** |  |  |  |  |  |
| Before sensitivity analysis | 15 | -0.59 (-2.56, 1.37) | 0.555 | 0.0% | 0.752 |
| After sensitivity analysis | 14 | 0.09 (-2.00, 2.17) | 0.935 | 0.0% | 0.927 |

HOMA-IR, homeostatic model assessment of insulin resistance; INS, insulin; FBG, fasting blood glucose.

Supplementary Table 8. Results of exploratory subgroup-specific sensitivity analyses.

| **Removed trials** | **No of studies** | **WMD (95%CI)** | ***P*-value** | **I^2^** | ***P*_heterogeneity_** |
| --- | --- | --- | --- | --- | --- |
| **HOMA-IR** |  |  |  |  |  |
| Excluding studies with concomitant lifestyle modification | 3 | 0.12 (-0.32, 0.56) | 0.578 | 0.0% | 0.584 |
| Including only studies with concomitant lifestyle modification | 7 | -0.68 (-1.08, -0.28) | **0.001** | 22.9% | 0.255 |
| Trials of purified hesperidin only | 5 | -0.89 (-1.28, -0.50) | **＜0.001** | 0.0% | 0.490 |
| Trials of hesperidin complexes only | 6 | 0.07 (-0.30, 0.45) | 0.699 | 0.0% | 0.761 |
| **QUICKI** |  |  |  |  |  |
| Excluding studies with concomitant lifestyle modification | 2 | 0.09 (-0.09,0.28) | 0.320 | 99.3% | **＜0.001** |
| Including only studies with concomitant lifestyle modification | 3 | 0.02 (0.02, 0.03) | **＜0.001** | 0/0% | 0.376 |
| **INS** |  |  |  |  |  |
| Excluding studies with concomitant lifestyle modification | 4 | 0.93 (-0.67, 2.53) | 0.256 | 0.0% | 0.647 |
| Including only studies with concomitant lifestyle modification | 7 | -2.79 (-4.30, -1.27) | **＜0.001** | 24.2% | 0.244 |
| Trials of purified hesperidin only | 7 | -3.22 (-4.64, -1.81) | **＜0.001** | 4.1% | 0.395 |
| Trials of hesperidin complexes only | 4 | 0.76 (-0.71, 2.23) | 0.312 | 0.0% | 0.737 |
| **FBG** |  |  |  |  |  |
| Excluding studies with concomitant lifestyle modification | 8 | 0.37 (-2.48, 3.21) | 0.801 | 0.0% | 0.953 |
| Including only studies with concomitant lifestyle modification | 7 | -1.47 (-4.19, 1.25) | 0.290 | 16.4% | 0.305 |
| Trials of purified hesperidin only | 10 | -0.01 (-2.47, 2.45) | 0.994 | 0.0% | 0.710 |
| Trials of hesperidin complexes only | 5 | -1.62 (-4.90,1.65) | 0.331 | 0.0% | 0.518 |
